# Experience Recruits MSK1 to Expand the Dynamic Range of Synapses and Enhance Cognition

**DOI:** 10.1523/JNEUROSCI.2765-19.2020

**Published:** 2020-06-10

**Authors:** Lucia Privitera, Lorenzo Morè, Daniel D. Cooper, Philippa Richardson, Marianthi Tsogka, Daniel Hebenstreit, J. Simon C. Arthur, Bruno G. Frenguelli

**Affiliations:** ^1^School of Life Sciences, University of Warwick, Coventry, CV4 7AL, UK; ^2^Centre for Discovery Brain Sciences, 1 George Square, Edinburgh EH8 9JZ, UK; ^3^School of Medicine, University of Dundee, Ninewells Hospital, Dundee, DD1 9SY, UK; ^4^School of Pharmacy and Biomedical Sciences, University of Central Lancashire, Preston, PR1 2HE, UK; ^5^School of Life Sciences, University of Dundee, Dundee, DD1 5EH, UK

**Keywords:** BDNF, cognition, environmental enrichment, MSK1, plasticity, transcriptomics

## Abstract

Experience powerfully influences neuronal function and cognitive performance, but the cellular and molecular events underlying the experience-dependent enhancement of mental ability have remained elusive. In particular, the mechanisms that couple the external environment to the genomic changes underpinning this improvement are unknown. To address this, we have used male mice harboring an inactivating mutation of mitogen- and stress-activated protein kinase 1 (MSK1), a brain-derived neurotrophic factor (BDNF)-activated enzyme downstream of the mitogen-activated protein kinase (MAPK) pathway. We show that MSK1 is required for the full extent of experience-induced improvement of spatial memory, for the expansion of the dynamic range of synapses, exemplified by the enhancement of hippocampal long-term potentiation (LTP) and long-term depression (LTD), and for the regulation of the majority of genes influenced by enrichment. In addition, and unexpectedly, we show that experience is associated with an MSK1-dependent downregulation of key MAPK and plasticity-related genes, notably of EGR1/Zif268 and Arc/Arg3.1, suggesting the establishment of a novel genomic landscape adapted to experience. By coupling experience to homeostatic changes in gene expression MSK1, represents a prime mechanism through which the external environment has an enduring influence on gene expression, synaptic function, and cognition.

**SIGNIFICANCE STATEMENT** Our everyday experiences strongly influence the structure and function of the brain. Positive experiences encourage the growth and development of the brain and support enhanced learning and memory and resistance to mood disorders such as anxiety. While this has been known for many years, how this occurs is not clear. Here, we show that many of the positive aspects of experience depend on an enzyme called mitogen- and stress-activated protein kinase 1 (MSK1). Using male mice with a mutation in MSK1, we show that MSK1 is necessary for the majority of gene expression changes associated with experience, extending the range over which the communication between neurons occurs, and for both the persistence of memory and the ability to learn new task rules.

## Introduction

Experience exerts a profound influence on the structure and function of the mammalian brain (Kolb and Whishaw, 1998). While this was predicted by early physiologists (von Bernhardi et al., 2017), it was the pioneering work of Donald Hebb in the 1940s that first demonstrated the enhanced cognitive abilities of rats raised in the stimulating environment of his home compared with their counterparts reared in a laboratory (Hebb, 1947, 1949). Subsequent studies have identified many cellular changes associated with the enhanced cognition that arises following exposure to larger social groups, a complex environment and exercise wheels, including neurogenesis, greater dendritic spine density and enhanced synaptic plasticity (Rosenzweig and Bennett, 1996; Sale et al., 2014; Ohline and Abraham, 2019). In experimental animals, these cellular adaptations translate into enhanced cognition (Sale et al., 2014), reduced anxiety (Rogers et al., 2019), the acceleration of recovery from brain injury (de la Tremblaye et al., 2019), resistance to drugs of abuse (Stairs and Bardo, 2009), and the alleviation of signs associated with animal models of Parkinson's disease (Wassouf and Schulze-Hentrich, 2019), Huntington's disease (Mo et al., 2015), and autism spectrum disorder (Gubert and Hannan, 2019). Indeed, parallels have been described in humans, including for children with autism (Woo and Leon, 2013; Woo et al., 2015; Aronoff et al., 2016), in terms of the benefits for mental health and wellbeing, and brain structure and function, of social interaction, skills training, exercise, and higher socioeconomic status (Kolb and Whishaw, 1998; Sale et al., 2014; Farah, 2018; Miguel et al., 2019; Rogers et al., 2019).

However, despite the importance of establishing how experience influences brain function, the intracellular signaling cascade and the enduring influence on the genome that underlie these cellular and cognitive adaptations to enriched environments have yet to be identified. An ideal candidate to mediate such a coupling between the environment and the genome would be positioned between the brain-derived neurotrophic factor (BDNF) TrkB receptors repeatedly implicated in mediating the benefits of environmental enrichment (Cowansage et al., 2010; Sale et al., 2014; Rogers et al., 2019), and the genomic changes required for persistent modifications to neuronal structure, synaptic function, and learning and memory (Alberini and Kandel, 2014; Takeuchi et al., 2014).

The nuclear kinase, mitogen- and stress-activated protein kinase 1 (MSK1), is well-placed to transduce the sensory experiences associated with enrichment into the enduring cellular, molecular and genomic events underpinning enhanced cognition. MSK1 is activated by BDNF and regulates gene expression, notably via the phosphorylation of cAMP response element-binding protein (CREB; Arthur et al., 2004; Reyskens and Arthur, 2016), and including that of the plasticity-related protein Arc/Arg3.1 (Hunter et al., 2017). In addition, MSK1 is expressed in hippocampal neurons (Heffron and Mandell, 2005; Sindreu et al., 2007), a major site of the effects of environmental enrichment (Hirase and Shinohara, 2014). Using mice harboring a knock-in point mutation of the MSK1 gene that results in the elimination of the kinase activity of MSK1 (kinase dead; *MSK1 KD*), but which does not affect hippocampal learning and memory or synaptic plasticity under standard housing conditions (Daumas et al., 2017), we previously showed that the kinase activity of MSK1 was required for homeostatic synaptic scaling *in vitro*, and the *in vivo* enrichment-induced enhancement of miniature EPSCs (mEPSCs; Corrêa et al., 2012; Lalo et al., 2018). However, this left unanswered the important question of the genomic, plasticity, and cognitive implications of these isolated observations at the synapse.

Using wild-type (WT) and *MSK1 KD* mice, we have found that the kinase activity of MSK1 is necessary for the full benefits of enrichment on cognition, in particular, in the persistence of hippocampal spatial memory and cognitive flexibility. As a potential cellular correlate of this enhanced cognition, we discovered that enrichment is associated with an MSK1-dependent expansion of the dynamic range of synapses: both hippocampal long-term potentiation (LTP) and long-term depression (LTD) are enhanced, thereby allowing synapses to code a greater amount of information. Finally, an RNA-Seq analysis of the hippocampal transcriptome under standard and enriched conditions revealed a predominant requirement for MSK1 in the experience-dependent regulation of gene expression. Moreover, we observed an unexpected and MSK1-dependent downregulation of plasticity-associated proteins and transcription factors such as Arc/Arg3.1 and EGR1. These observations suggest that MSK1 couples the external environment to the genome, and through this coupling initiates both the cellular and molecular events leading to synaptic and cognitive enhancement, and an experience-dependent genomic homeostasis designed to maintain the stability of the enhanced brain.

## Materials and Methods

### Animals

The *MSK1 KD* mouse used in this study has been described previously (Corrêa et al., 2012). Briefly, Asp194 in the endogenous MSK1 gene was mutated to Ala (D194A). This inactivates the N-terminal kinase domain of MSK1. Genotyping was conducted by PCR using the primers 5′-CGGCCATGTGGTGCTGACAGC-3′ and 5′-GGGTCAGAGGCCTGCACTAGG-3′, which gives 378- and 529-bp products for WT and targeted alleles, respectively. All the mice used in this study were on a C57-Bl/6J genetic background after at least four backcrosses from the original C57-Bl/6n strain used by Taconic Artemis to generate the mutant mice. Male WT C57-Bl/6J mice purchased from Charles River UK were used for backcrossing with female *MSK1 KD* homozygous mutants. The mice used in this study were maintained as homozygous and WT lines derived from founder homozygous and WT breeders from an initial series of heterozygote crosses. Subsequent backcrossing occurred when the founder mice had come to the end of their reproductive lifetime (typically three litters). This strategy avoided genetic divergence of the two lines. While using WT and homozygous mutant littermates from heterozygote crosses is experimentally desirable, our breeding strategy is appropriate when homozygous mutants of both sexes are viable and fertile (Jax, 2009), allowed large numbers of animals of the correct age, genotype, housing condition, and sex to be bred in order that experiments could be conducted in time-limited batches, minimizing variability. Our breeding strategy also avoided the unnecessary breeding and culling of large numbers of heterozygote mice (50% of all litters) in keeping with the drive to reduce the number of animals used in research, and with institutional and funder expectations. We note that many experimental parameters were similar between WT and *MSK1 KD* mice under standard and enriched housing conditions, and that the hippocampal expression of only three genes differed between the two genotypes under standard housing conditions. This suggests that the breeding strategy did not introduce confounds that could have affected our observations.

Mice were maintained under a 12/12 h light/dark cycle (lights on at 7 A.M.) in a facility kept at 20–24°C and were given *ad libitum* access to standard mouse chow and water. All animal procedures conformed with local, national, and EU guidelines concerning the welfare of experimental animals. Behavioral studies were performed under the auspices of Home Office license PPL 70/7821 granted to B.G.F. Male mice were used in this study to facilitate comparison with previous studies on *MSK1 KD* mice (Corrêa et al., 2012; Daumas et al., 2017). The mice have been deposited with the INFRAFRONTIER/EMMA repository at MRC Harwell Institute.

### Environmental enrichment

Environmental enrichment was provided via the rearing of WT and *MSK1 KD* mice in large individually ventilated rat cages (Tecniplast 1500U; 480 × 375 × 210 mm; 1500-cm^2^ floor area) containing bedding material, a cardboard tube, one running wheel and several plastic toys (tunnels, platforms, seesaws) and a metal ladder. To provide novelty, toys were moved around twice per week and new toys introduced once per week. Two to three pregnant dams (with pregnancies at embryonic (E) days E14–E15, based on vaginal plugs) were randomly selected and placed in enriched cages to provide additional mothering (D'Amato et al., 2011) and larger groups for social interactions. Dams typically gave birth within 1–2 d of each other. At weaning (postnatal (P) day P23–P24), all females were removed and the males (typically eight) remained in the enriched environment for the remainder of the experimental period (to approximately five months of age). Age-matched male mice were born and maintained in standard housing (Tecniplast 1284L; 365 × 207 × 140 mm; 530-cm^2^ floor area; two to four mice with bedding material and a cardboard tube) and served as controls for the environmental enrichment groups. Cage cleaning was done on Mondays for all standard and enriched cages. Toys in enriched cages were changed on Tuesdays and were moved around the enriched cages on Mondays and Thursdays. To keep disruption of the home environment to a minimum, sawdust and bedding were never changed at the same time as toys. To minimize disruptions to established hierarchies, during cage cleaning and behavioral testing all mice (standard and enriched) were removed to a different cage (standard cage size with one toy from the enriched cage for enriched mice) and then were returned together. This was effective in reducing within-cage aggression between the males.

### Analysis of dendritic spine density

Male WT and *MSK1 KD* mice were killed at 17–18 weeks of age, two weeks after the end of behavioral testing, by cervical dislocation in accordance with the United Kingdom Animals (Scientific Procedures) Act 1986 and with local Animal Welfare and Ethical Review Board approval. The brains were removed and processed with the FD Rapid Golgi Stain kit (FD NeuroTechnologies) in accordance with the manufacturer's protocol. Impregnated brains (four per group) were sectioned with a vibratome (coronal sections; 200 µm thick) stained and mounted. Dendritic spines on the secondary branches of apical dendrites of hippocampal CA1 neurons were counted. Spine count was conducted blind to genotype and housing condition. ImageJ software was used to measure dendritic length and the numbers of spines on each dendritic segment. Images for spine density analysis were captured with a 40× objective on a Zeiss Imager 2 AXIO microscope.

### Hippocampal slice preparation and extracellular recordings

Male WT and *MSK1 KD* mice (three to five months old) were killed by cervical dislocation in accordance with the United Kingdom Animals (Scientific Procedures) Act 1986 and with local Animal Welfare and Ethical Review Board approval. Hippocampal slices (400 µm) were cut in ice cold aCSF using either a Stoelting tissue chopper or a Microm HM650V tissue slicer. Upon cutting, slices were transferred to a recording chamber and placed on a mesh support at the interface of an oxygen-rich atmosphere and underlying aCSF where they remained for the duration of the experiment, which typically started some 2 h after slice cutting. The temperature of the aCSF was set at 31°C and the flow rate was 1.5 ml/min. The aCSF used for the preparation, maintenance and recording of slices contained the following: 124.0 mm NaCl, 4.4 mm KCl, 1.0 mm Na_2_HPO_4_, 25.0 mm NaHCO_3_, 2.0 mm CaCl_2_, 2.0 mm MgCl_2_, and 10.0 mm D-glucose. aCSF was bubbled with 95% O_2_/5% CO_2_, pH 7.4. All salts used in the aCSF were obtained from either Fisher Scientific or Sigma-Aldrich.

To make extracellular recordings of field EPSPs (fEPSPs), an aCSF-filled glass microelectrode was placed in stratum radiatum of area CA1 and two concentric bipolar stimulating electrodes (CBBRC75, FHC) were placed either side of the recording electrode. This allowed alternating recordings to be made from two independent but convergent afferent Schaffer collateral/commissural fiber pathways. Each pathway was stimulated every 90 s with a monophasic pulse of 0.1-ms duration. Pathway-independence was assessed via a crossed paired-pulse facilitation (PPF) protocol (at 50 ms interpulse interval). Independence was accepted when facilitation of the second pulse was ∼10% or less. To assess basal synaptic transmission, stimulus input/fEPSP slope output curves were constructed over the range of 20–300 µA. A minimum of four fEPSPs were averaged to yield a fEPSP slope measurement at each stimulus intensity. At the highest stimulus intensity (300 µA), and where visible, the presynaptic fiber volley was measured as an indicator of the recruitment of afferent axons. PPF, a commonly used index of the probability of neurotransmitter release, was assessed over an interstimulus interval of 50–350 ms, with the average of at least two fEPSPs yielding the slope measurement at each paired-pulse interval. In all experiments both pathways in each slice were tested for input-output and PPF profiles and all were taken into consideration in subsequent analyses.

For the LTP and LTD experiments, a stable baseline of at least 30 min was achieved before theta-burst (TBS) or low-frequency stimulation (LFS) was delivered to one pathway. TBS consisted of bursts of four stimuli at 100 Hz with 10 such bursts comprising a train. Each burst within a train was separated by 200 ms. Trains were repeated three times with an intertrain interval of 20 s. LFS consisted of 900 pulses at 1 Hz. The second pathway was not subject to TBS or LFS and served as a control for the stability of the recordings. Experiments were excluded from analysis if the control pathway deteriorated by >10% within the 3 h post-TBS, and 1 h post-LFS, monitoring period.

Given the deficit in basal synaptic transmission observed in *MSK1 KD* mice, care was taken to match the baseline strength of synaptic transmission, which involved adjusting the stimulus intensity to yield fEPSPs of ∼3 mV across all groups. A one-way ANOVA showed no difference in baseline fEPSP amplitudes across the four experimental groups for either the LTP (*F*_(3,28)_ = 0.676, *p* = 0.574) or LTD (*F*_(3,22)_ = 0.975, *p* = 0.422) experiments. Electrophysiological recording parameters and the analysis of fEPSPs were under the control of WinLTP program (Anderson and Collingridge, 2007). LTD experiments, and the majority of LTP experiments, were performed in experimentally naive mice of three to four months of age. Experiments were interleaved and performed blind to the identity and housing condition of the mice, which was revealed only after the experiments had been analyzed and genotype confirmed with *post hoc* genotyping as required.

### Western blotting

Experimentally-naive mice (three to four months of age) were killed by cervical dislocation as described above (Hippocampal slice preparation and extracellular recordings). The brain was removed and individual hemispheres were snap-frozen in liquid nitrogen and stored at −80°C. When required, samples were defrosted, the hippocampus dissected free and lysed in lysis buffer containing the following: 50 mm Tris-HCl (pH 7.5), 1% Triton X-100, 0.1% SDS, 1 mm Na_3_VO_4_, 50 mm NaF, 5 mm Na_4_P_2_O_7_, 0.27 M sucrose, 0.02% NaN_3_, and protease inhibitor mixture tablets (Roche). The tissue underwent mechanical disruption using a Dounce homogenizer. Samples were then stored on ice before rotation at 4°C for 30 min, followed by centrifugation for 20 min and 12,000 × *g* at 4°C. The protein concentration of each sample was calculated using a standard BCA curve. Samples were aliquoted, mixed with loading buffer, and stored at −20°C until required for western blotting.

After defrosting, samples were brought to 80°C for 5 min, spun briefly and the proteins were separated using SDS-PAGE electrophoresis in an 8% gel. After separation, proteins were transferred onto nitrocellulose blotting membrane (GE Healthcare) in a semi-wet system for 2.5 h at 200 µA. The membrane was blocked in 10% Marvel milk powder and 0.5% Tween for 1 h. Membranes were incubated in glyceraldehyde-3-phosphate dehydrogenase (GAPDH) primary antibody (Table 1) in 1% milk powder 0.05% PBS Tween (PBS-T) solution for 2 h at room temperature, following which they were washed for 10 min four times in 0.1% PBS-T. Samples were incubated overnight at 4°C with a second primary antibody (GluA1, GluA2, EGR1, or Arc/Arg3.1; Table 1) then washed four times for 10 min in PBS-T. Membranes were incubated for 1–2 h in horseradish peroxidase (HRP)-conjugated anti-rabbit antibody (1:10,000 dilution; ThermoFisher #31460). After four 10 min washes with PBS-T, membranes were incubated for 2 min in Clarity Western ECL Substrate (Bio-Rad) and imaged using the Image Quant LAS 4000 CCD biomolecular imager. Image Studio Lite vs 5.2.5 was used to analyze the signal of the bands and the protein of interest was normalized to GAPDH. Control blots confirmed that the GAPDH antibody gave no detectable bands at the predicted molecular weights of GluA1, GluA2, EGR1, or Arc/Arg3.1.

**Table 1.** Antibodies used in the study

Primary antibody	Supplier (catalog #)	Concentration of primary Ab
EGR1	CST (4153S)	1:500
Arc/Arg3.1	Abcam (AB183183)	1:500
GluA1	Abcam (AB31232)	1:1000
GluA2	Merck (AB 1768)	1:2000
GAPDH	CST (2118S)	1:160,000 (for Egr1 or Arc/Arg3.1 blots)
		1:40,000 (for GluA1 or GluA2 blots)

### Behavioral procedures

Mice used were male aged 2.5–3.5 months and were scored for weight and against a battery of tests for neurological signs (Wolf et al., 1996) before any behavioral experiment began. No neurological signs were observed across any of the groups (data not shown). Different tests were conducted at weekly intervals to avoid one test influencing another: the week commencing (w/c) P70 open field and novel object; w/c P77 elevated plus maze; w/c P84 spontaneous alternation; w/c P91–w/c 104 water maze, a two-week protocol.

#### Open field and novel object

These tests were run as two consecutive stages of the same experiment. Four open field boxes (Ugo Basile; 44 × 44 × 44 cm) were placed inside the empty water maze arena to form a square. Four mice were tested simultaneously. Each mouse was singly released in each box and tracked. Exposure to the open field lasted for 1 h after which, for the novel object stage of testing, a 50-ml plastic vial (Falcon) was secured upside-down to the center of the arena and the mouse was tracked for an additional hour.

#### Elevated plus maze

An eight-radial arm maze for mice (Ugo Basile) was placed within the empty water tank and raised 60 cm from the tank base. Four of the eight arms were kept open to form a plus shape; two of the arms had walls while the other two (opposite one another) were without walls. Each mouse was individually released in the center of the maze and video tracked for 10 min.

#### Spontaneous alternation for spatial working memory

An eight-radial arm maze for mice (Ugo Basile) was placed within the circular confines of the tank used for the water maze. Four out the eight arms (with walls) were kept open to form a cross. The entrances to the other four arms were closed. Each mouse was individually released in the center of the maze and video tracked for 10 min. The sequence of arm entries was scored. A correct alternation was considered when a mouse made no repetition in four entries (Mohler et al., 2007).

#### Water maze for spatial reference memory

Experimentally-naive mice were used and an intertrial interval of 120 s over four daily trials was employed. The pool was filled daily with fresh water, which was made opaque by the use of UHT milk.

##### Stage 1, habituation

Each mouse was placed on a 20 cm diameter platform located in the center of a 180 cm diameter pool filled with opaque water (28°C) and was allowed to observe the environment for 2 min. The pool was surrounded by curtains which did not allow the distal visual cues to be seen. Water level was ∼1 cm above the top of the platform. Each mouse then received three consecutive trials (different starting points) where it was left free to swim in the pool for a maximum of 2 min and then placed on the platform and left there for 30 s.

##### Stage 2 (days 2 and 3), visual cue

The platform was placed in the center of the pool and a visible object was placed on it (yellow TV toy 6 × 6 × 5 cm). Each mouse received four consecutive trials (different cardinal starting points) where it was left free to swim in the pool for a maximum of 2 min. Water level was ∼1 cm above the platform surface. Water was kept at 26°C. The pool was surrounded by curtains which did not allow the distal visual cues to be seen.

##### Stage 3 (days 4–7), training

Curtains were removed. Water was kept at 26°C. The platform was placed in the center of the South-East or North-West quadrant and kept constant for any given mouse. Water level was ∼1 cm above the platform surface. Each mouse received four trials (different starting points) where it was left free to swim in the pool for a maximum of 2 min and then left on the platform for 30 s.

##### Stage 4 (day 8), precision testing

The platform was reduced from 20 to 10 cm in diameter to test for more specific memory of the location of the escape platform. All other parameters as per stage 3.

##### Stage 5 (day 9), 24 h delay probe trial

Water was kept at 26°C. The platform was removed and distal spatial cues were present as per previous the stage. Each mouse received a single 120 s trial. Starting point was distal to the location of the platform during training, e.g., if platform was South-East, starting point was North.

#### Water maze reversal learning protocol for cognitive flexibility

##### Stage 1, habituation

As described above (Water maze for spatial reference memory), but 2 d were given instead of one.

##### Stage 2 (days 3 and 4), visual cue

As described above.

##### Stage 3 (days 5–7), training

As described above.

##### Stage 4 (days 8 and 9), reversal learning

The platform was placed in the quadrant opposite to that used during training. All other parameters as per stage 3.

##### Stage 5 (day 10), 24 h delay probe trial

As described above. Starting point was distal to the location of the platform during reversal learning stage (Stage 4).

Mice used for this experiment were experimentally-naive with respect to the water maze but had undergone open field ± novel object, the elevated plus maze, and spontaneous alternation.

Behavioral tests were video-tracked and analyzed using AnyMaze 4.99 video-tracking system. All the behavioral experiments were conducted blind to genotype.

### RNA-Seq

Hippocampal RNA was prepared from experimentally-naive mice of three to four months of age. Samples were prepared and analyzed blind to the two genotypes and two housing conditions. The hippocampi from contemporaneous mice were used for western blotting for GluA1, GluA2, EGR1, and Arc/Arg3.1 as described above (Western blotting).

#### RNA extraction and library preparation

Hippocampi were extracted and then rapidly homogenized in TRIzol (Invitrogen, #15596018). Total RNA was precipitated using isopropanol following the manufacturer's protocol and treated with DNaseI. RNA quality was checked using Nanodrop and a Qubit 4 fluorimeter (Invitrogen).

mRNA libraries were prepared using the TruSeqv2 (Illumina, #RS-122-2001) LS protocol in-house by the School of Life Sciences Genomics Facility. Briefly, poly-A mRNA was pulled down using poly-T magnetic beads, fragmented, and primed with random hexamers before first-strand synthesis. Following second-strand synthesis, blunt end repair were performed with a 3′ to 5′ exonuclease, and 3′ ends adenylated. Adaptors were then ligated to the cDNA. All 24 library samples were quality checked on a 2100 bioanalyser (Agilent) and assayed on a Qubit 4 fluorimeter (Invitrogen) before being multiplexed 6 samples to a lane and sequenced at 150-bp paired end on an Illumina HiSeq 4000. An average of 41.76 million reads per sample were obtained (Extended Data Table 7-1).

10.1523/JNEUROSCI.2765-19.2020.t7-1 Table 7-1. Supplementary Multimedia/Extended Data. Download Table 7-1, XLSX file

#### Analysis pipeline

##### Quality control and trimming

Samples were de-multiplexed and the raw fastq files quality checked using FastQC (v0.11.3; Andrews, 2010). Adaptor contamination was removed using Skewer (v0.2.2; Jiang et al., 2014), with Illumina TruSeq v2 adapter lists, including reverse complements and theoretical PCR product. Fastq files were also trimmed if the mean quality of bases dropped below 10 (4 bp window), and only reads >50 bp were kept. Adapter contamination removal was confirmed using FastQC. Paired fastq files for each sample (forward and reverse) were aligned to the mouse genome (GRCm38) using STAR aligner (v2.5; Dobin et al., 2013), and annotated (GRCm38.87). Average read alignment was 92.29% after exclusion of the two samples that failed quality control (Extended Data Table 7-1). Aligned BAM files were then loaded into IGV (v2.3.65; Robinson et al., 2011) and compared at the MSK1 gene locus, to check for a mismatch in the kinase domain of the MSK1 gene introduced as a point mutation into the KD mutants (Corrêa et al., 2012). QC metrics were calculated for each sample using SeqMonk (Andrews, 2018), examining high probe read counts across ribosomal RNA (rRNA) and mitochondrial genes and observing how many reads fell within genes and exons. HtSeq (v0.6.1p1; Anders et al., 2015) was then used to quantify read counts for individual genes, using default parameters, specifying unstranded reads and only unique read alignment. A minimum average PHRED quality score of 10 was necessary for reads to be counted.

Sample 34B contained a large percentage (∼33%) of reads mapping to rRNA. Additionally, sample 34B contained ∼1% of the read number of sample 35A, indicating poor amplification of the cDNA library, and contained unacceptable sequence duplication levels. Ribosomal (rRNA) contamination (∼25% of reads) was also observed in sample 33B. Both sample 34B and 33B were removed from further analysis based on poor quality control metrics.

Intragroup sample variation was observed to be quite high for some samples, and this interfered with obtaining good quality distinct expression. Therefore, several more samples were removed for each group, based on how well they correlated with other within-group samples. After alignment QC, and after removing samples displaying poor intragroup correlation, a new sample table was made for testing differential gene expression (Extended Data Table 7-2). Differential gene expression lists for each condition comparison are included in Extended Data Table 7-3.

10.1523/JNEUROSCI.2765-19.2020.t7-2 Table 7-2. Supplementary Multimedia/Extended Data. Download Table 7-2, XLSX file

10.1523/JNEUROSCI.2765-19.2020.t7-3 Table 7-3. Supplementary Multimedia/Extended Data. Download Table 7-3, XLSX file

Principal component analysis was conducted in R (3.5.0; RTeam, 2018) using the DEseq2 package (v1.20.0; Love et al., 2014). Differential gene expression statistical comparisons were conducted using the Wald test statistic with a Benjamini–Hochberg-corrected *p* value cutoff of ≤0.05, and a log2 fold-change cutoff of 0.38 (corresponds to a 1.3-fold increase/decrease) used to define significant differentially expressed genes. Gene ontology (GO) enrichment analysis was performed using the topGO package (v2.32.0; Alexa and Rahnenfuhrer, 2006) using the “classic” algorithm (Alexa and Rahnenfuhrer, 2006) and Fisher's exact test for enrichment scoring against the ontology org.Mm.eg.db (v3.6.0; Carlson, 2018). Multiple-testing correction was conducted using Benjamini–Hochberg correction, and GO terms were considered significant at a corrected *p* ≤ 0.01. Unless explicitly stated, default parameters were used for all tools, and scripts used for these analyses and will be provided on request. The RNAseq data in this publication have been deposited in NCBI's Gene Expression Omnibus and are accessible through GEO Series accession number GSE149210.

### Statistical analysis

Statistics were computed by IBM SPSS 25 using two-tailed one-way or two-way ANOVA with genotype and housing condition as the two between group factors and day of training, time point, or stimulus strength as within factor as appropriate, with simple main effects or main effects as the *post hoc* comparison. In the absence of significant housing × genotype interactions, planned comparisons regarding the effects of genotype or housing were conducted, in keeping with the views expressed in expert treatments of statistics (Faraway, 2005; Kutner et al., 2005; Howell, 2010; Laerd Statistics, 2017; Wei et al., 2012). The level of significance was taken to be *p* < 0.05. Data are reported as mean ± SEM, and bar graphs display individual data points.

## Results

### MSK1 is necessary for the full extent of experience-dependent enhancement of cognition

**Figure 1.**
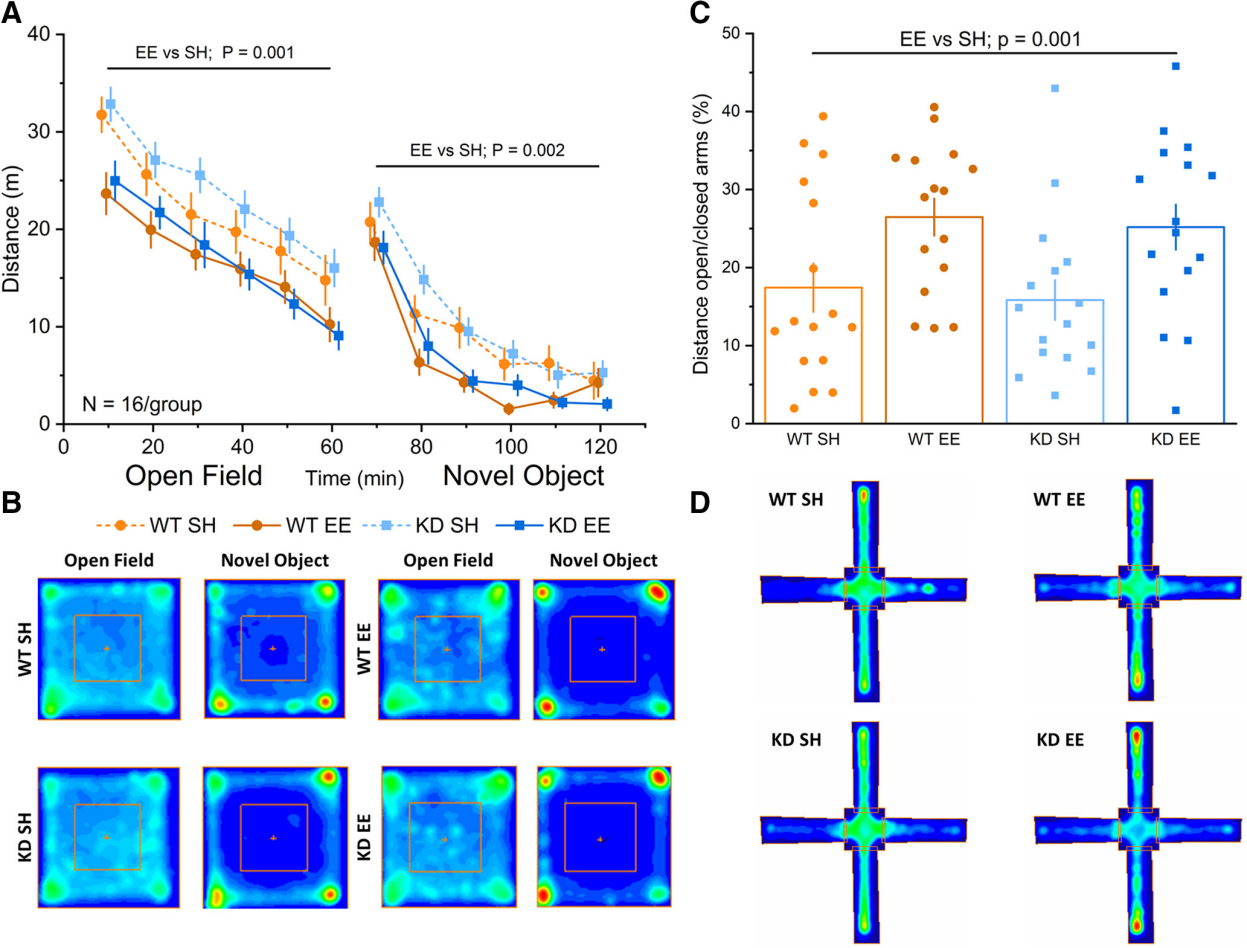
Environmental enrichment influences exploration and anxiety independently of MSK1. ***A***, Mice (*n* = 16 per group) of both genotypes (WT and *MSK1 KD*) and housing condition (standard housed (SH) and enriched (EE)) were placed in an open field for 60 min (left half of graph) after which a 50 ml plastic tube was introduced into the arena (right half of graph; novel object). Animal locomotion was tracked for the entire period. During the first 60 min, a RM-ANOVA showed an effect of time for all groups (*F*_(5,300)_ = 149.89, *p* = 0.001) indicating a reduction in activity over this period, and also a housing effect (*F*_(1,60)_ = 12.10, *p* = 0.001) indicating that standard housed mice were more active regardless of their genotype. During the second 60 min of the test (after the introduction of the novel object) a RM-ANOVA showed an effect of time for all groups (*F*_(5,300)_ = 220.46, *p* = 0.001) indicating a reduction in activity over time, and also a housing effect (*F*_(1,60)_ = 10.31, *p* = 0.002) showing that standard housed mice remained more active in response to the introduction of a novel object regardless of their genotype. Data are presented as mean ± SEM. Data points have been offset for clarity. ***B***, Heat maps of activity before and after introduction of the novel object for each of the four groups of animals. ***C***, Mice (*n* = 16 per group) were placed on the central area of an elevated plus maze and allowed to explore for 10 min during which time their activity was video-tracked. Enriched mice of either genotype traveled further in the open arms compared with mice housed under standard conditions (*F*_(1,60)_ = 11.21 *p* = 0.001). Bars show the mean ± SEM and individual data points. ***D***, Occupancy heat maps for each of the groups, with the closed arms of the elevated plus maze running vertically.

To confirm that our enrichment protocol had tangible effects on animal behavior, we initially assessed the influence of enrichment on locomotor function and anxiety, the latter of which in particular is sensitive to enrichment (Rogers et al., 2019). WT and *MSK1 KD* mice raised from birth in standard housing behaved similarly when exposed to an open field arena (Fig. 1*A*,*B*) and the elevated plus maze (Fig. 1*C*,*D*). In contrast to their counterparts raised in standard housing, enriched animals of both genotypes displayed reduced locomotor activity in the open field and in response to the introduction of a novel object (Fig. 1*A*,*B*) and traveled further in the open arms of the elevated plus maze, indicative of reduced anxiety (Fig. 1*C*,*D*). These observations confirm the effectiveness of the enrichment protocol in influencing behavior, the absence of gross sensorimotor impairments in the *MSK1 KD* mutant mice that could confound subsequent investigations, for example, due to the high levels of MSK1 in the striatum and cerebellum (Heffron and Mandell, 2005), and the ability of *MSK1 KD* mice to display some benefits of enrichment.

**Figure 2.**
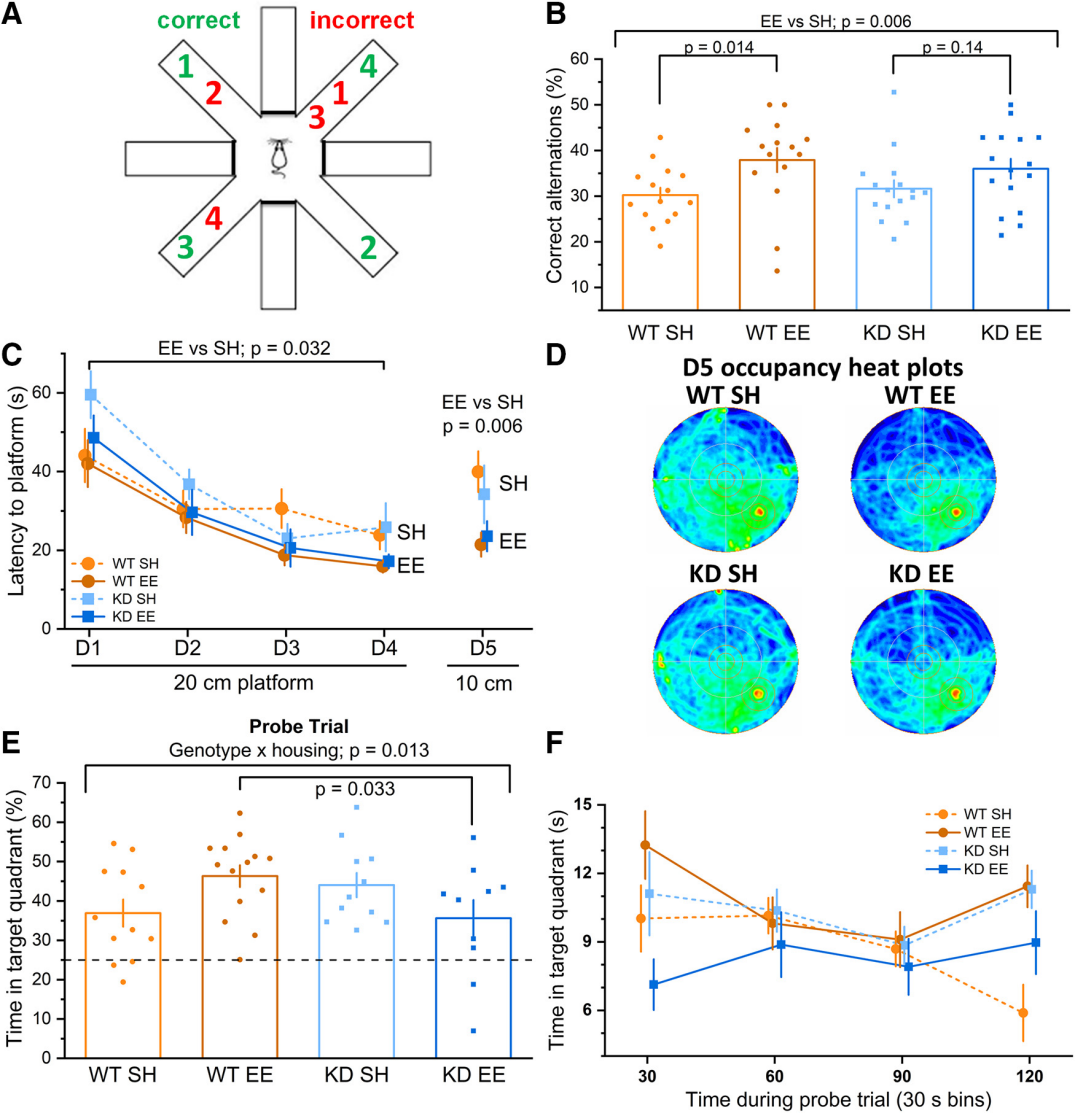
The kinase activity of MSK1 is necessary for enrichment-induced memory persistence ***A***, In a spontaneous alternation test of spatial working memory, mice (*n* = 15–16 per group) were exposed for 10 min to a radial maze (with walls) in which four arms were open to the mice. The mice scored correctly if four different arms were visited before a return visit occurred to any one of the arms. ***B***, The percentage of correct alternations was higher for enriched mice (EE) regardless of genotype (housing effect: *F*_(1,58)_ = 8.15, *p* = 0.006). While there was no significant genotype × housing interaction, the difference between enrichment and standard housed (SH) mice was greater in the WT, which showed a significant improvement (*F*_(1,58)_ = 6.41, *p* = 0.014), compared with the *MSK1 KD* (KD) that showed no improvement (*F*_(1,58)_ = 2.22, *p* = 0.14). Bar graph depicts mean ± SEM and individual data points. ***C***, Mice (*n* = 10–14 per group) were trained on the water maze over five consecutive days. All groups showed learning of the platform location over the first stage of training (4 days; D1–D4) with a 20 cm platform. A RM-ANOVA, with genotype and housing as factors, showed a significant effect of session (*F*_(3,129)_ = 36.16, *p* = 0.000) on the latency to reach the escape platform. It also showed a main effect of housing (*F*_(1,43)_ = 4.94, *p* = 0.032) where enriched mice of both genotypes performed better than standard housed mice, with no difference in performance between standard housed mice. On the second stage of the training (1 days; D5), in which a 10 cm platform was employed to test for more precise spatial reference memory, the ANOVA on the latency to reach the escape platform with between factors as genotype and housing again showed an effect of housing with better performance in enriched mice (*F*_(1,43)_ = 8.45, *p* = 0.006). Data shown as mean ± SEM. Data points are offset for clarity. ***D***, Heat maps of trajectories for all four groups. The platform was located in the south east quadrant. More selective searching (greater occupancy of the training quadrant) can be seen in the enriched mice. ***E***, On the probe trial 24 h after D5, enriched WT mice showed the best retention for the spatial location of the escape platform, whereas the enriched *MSK1 KD* mice showed the worst performance. The ANOVA on the total time spent on the target quadrant (where the platform had been previously) showed a significant interaction between genotype and housing (*F*_(1,43)_ = 6.65, *p* = 0.013), and the simple main effect showed a significant difference between enriched WT mice and enriched *MSK1 KD* mice (*F*_(1,43)_ = 4.84, *p* = 0.033). Bar graph depicts mean ± SEM and individual data points. ***F***, The retention deficit in the enriched *MSK1 KD* mice was observed across the entire probe trial duration where they spent consistently less time in the appropriate quadrant compared with other groups. Data shown as mean ± SEM. Data points are offset for clarity.

To assess hippocampus-dependent forms of learning and memory, we began by testing spatial working memory using a spontaneous alternation task (Fig. 2*A*). While standard housed mice of both genotypes performed at comparable levels (Fig. 2*B*), there was a significant effect of enrichment that was reflected in significantly improved performance in WT mice, but not in the *MSK1 KD* animals (Fig. 2*B*).

To extend these observations to spatial reference memory, the four groups of mice were tested in the Morris water maze. Both *MSK1 KD* and WT mice benefitted from enrichment in learning the position of the platform more rapidly than their standard housed counterparts, who performed comparably, as described previously (Daumas et al., 2017; Fig. 2*C*). Similarly, when the20 cm platform was replaced with a 10 cm platform on day 5 of training to test the accuracy of learning the location of the platform, enriched mice of both genotypes were better able to locate the smaller diameter platform than standard housed mice, with the more selective searching of the enriched mice obvious from heat maps of trajectories obtained on day 5 (Fig. 2*D*). These data suggest that *MSK1 KD* mice can display some cognitive benefits of enrichment. However, these benefits were not lasting over time: during the probe trial 24 h later, enriched WT mice spent significantly more time in the training quadrant than their standard housed counterparts, while no enrichment-induced improvement was observed in the *MSK1 KD* mice (Fig. 2*E*,*F*). MSK1 is therefore required for the full extent of enrichment-induced persistence of spatial reference memory.

**Figure 3.**
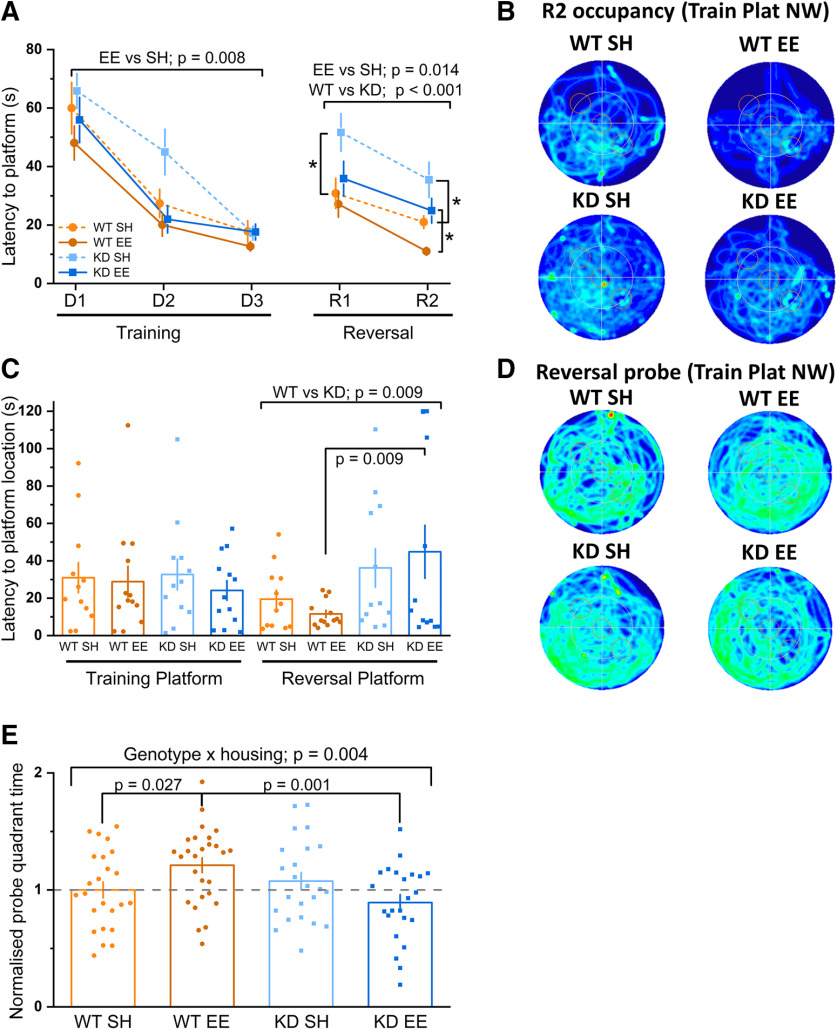
The kinase activity of MSK1 is necessary for enrichment-dependent cognitive flexibility and long-term memory ***A***, All groups (*n* = 12 or 13 per group) showed learning over the first stage of training on the Morris water maze (3 days; D1–D3). A RM-ANOVA with genotype and housing as between factors showed a significant effect of session (*F*_(2,92)_ = 71.91, *p* = 0.000) on the latency to reach the escape platform. It also showed a main effect of housing (*F*_(1,46)_ = 7.76, *p* = 0.008) where the enriched mice performed better than standard housed mice. On the reversal learning stage (2 days; R1–R2), a RM-ANOVA on the latency to reach the new escape platform location showed an effect of session (*F*_(1,46)_ = 16.15, *p* = 0.000), genotype (*F*_(1,46)_ = 15.66, *p* = 0.000), and housing (*F*_(1,46)_ = 6.59, *p* = 0.014) indicating that all groups learned over the 2 days, but WT mice performed better than *MSK1 KD* mutant mice, and enriched mice better than standard housed mice. There was no significant interaction between genotype and housing, but on the first day of reversal learning (R1), there was a significant difference between standard housed WT mice and standard housed *MSK1 KD* mice (*; *F*_(1,46)_ = 5.54, *p* = 0.023). On R2, there was a significant difference between enriched WT mice and enriched *MSK1 KD* mice(*; *F*_(1,46)_ = 6.42, *p* = 0.015) and again between standard housed WT mice and standard housed *MSK1 KD* mice (*; *F*_(1,46)_ = 6.32, *p* = 0.016). Data shown as mean ± SEM. Data points are offset for clarity. ***B***, Occupancy plots for the second day of reversal learning (R2), which show preferential searching in the reversal quadrant (south east) by enriched WT mice. ***C***, On the probe trial 24 h later, enriched WT mice showed the best goal-directed behavior for the reverse location of the escape platform, while the enriched *MSK1 KD* mice again showed the worst performance. While there was no significant interaction between genotype and housing, an ANOVA on the latency to enter the reversal location of the platform showed a significant effect for genotype (*F*_(1,46)_ = 7.51, *p* = 0.009) and also a significant difference between enriched WT and enriched *MSK1 KD* mice (*F*_(1,46)_ = 6.39, *p* = 0.015). Bar graph depicts mean ± SEM and individual data points. ***D***, inset, Heat maps of occupancy in the water maze during the 120 s probe trial for the four groups. Enriched WT mice show activity concentrated in the reversal learning quadrant (south east). ***E***, A comparison of 24-h probe trial performance across the two experiments [small platform (Fig. 2*E*,*F*) and reversal learning (***C***, ***D***)], where performance across groups was normalized to that of WT standard housed mice, showed a significant genotype × housing interaction (*F*_(1,93)_ = 8.49, *p* = 0.004), where enriched *MSK1 KD* mice performed significantly worse that their enriched WT counterparts (*F*_(1,93)_ = 11.48, *p* = 0.001), who in turn performed better than standard housed WT mice (*F*_(1,93)_ = 5.08, *p* = 0.027). Data from 23–27 mice per group. Broken line indicates level of performance when normalized to the respective WTSH mean.

To further probe the requirement for MSK1 in the cognition-enhancing effects of enrichment, new experiments with four additional groups of water maze-naive animals were conducted using the Lipp/Wolfer protocol for cognitive flexibility (Lipp and Wolfer, 1998). After training to asymptotic levels by day 3, the location of the escape platform was moved to the opposite quadrant and performance assessed on days 4 [reversal (R) day 1 (R1)] and 5 (R2; Fig. 3*A*). As expected, switching the location of the platform to the opposite quadrant resulted in longer escape latencies across all groups on the first exposure on R1 (Fig. 3*A*). However, by the second day of platform reversal (R2), the enriched WT mice escaped more quickly than standard housed WT mice. This pattern was not seen in enriched *MSK1 KD* mice, with latency to platform and heat maps of their swimming trajectory observed to be comparable to that of standard housed WT mice (Fig. 3*A*,*B*). This suggests a requirement for MSK1 in the cognitive flexibility required for reversal learning.

Moreover, the failure to retain information over 24 h was again shown by MSK1 mutant mice, but now concerning the new location for escape: in the probe trial given 24 h later (Fig. 3*C*,*D*), both standard housed and enriched WT mice showed clear preference to navigate to the most recent (reversed) location of the platform. In contrast, MSK1 mutant mice either displayed no preference between the new and the old escape locations or, in the case of the enriched *MSK1 KD* mice, even a preference for the former location of the platform used for training on days 1–3 (Fig. 3*C*,*D*).

*MSK1 KD* mice that had experienced enrichment seemed to fare worse on both probe trial tests for memory persistence. To examine this in greater detail, we considered the amount of time spent in the appropriate quadrant for both the small platform experiment (target quadrant; Fig. 2*C–F*) and the reversal learning experiment (reversal quadrant; Fig. 3*A–D*). To compensate for differences in performance across these two trials, we normalized performance across all groups to the mean of the percentage time spent in the appropriate quadrant by standard housed WT mice. Bringing these two datasets together in this way increases the power of the observations and allows more robust conclusions to be drawn as to the performance of enriched *MSK1 KD* mice. This analysis (Fig. 3*E*) showed a significant genotype × housing interaction with enhanced memory ability in enriched WT mice compared with enriched *MSK1 KD* mice. These observations indicate that enrichment improves both spatial working and reference memory in WT mice and that both the persistence of memory and reversal learning, an index of cognitive flexibility, requires the kinase activity of MSK1.

### Environmental enrichment induces synaptic homeostasis in an MSK1-dependent manner

**Figure 4.**
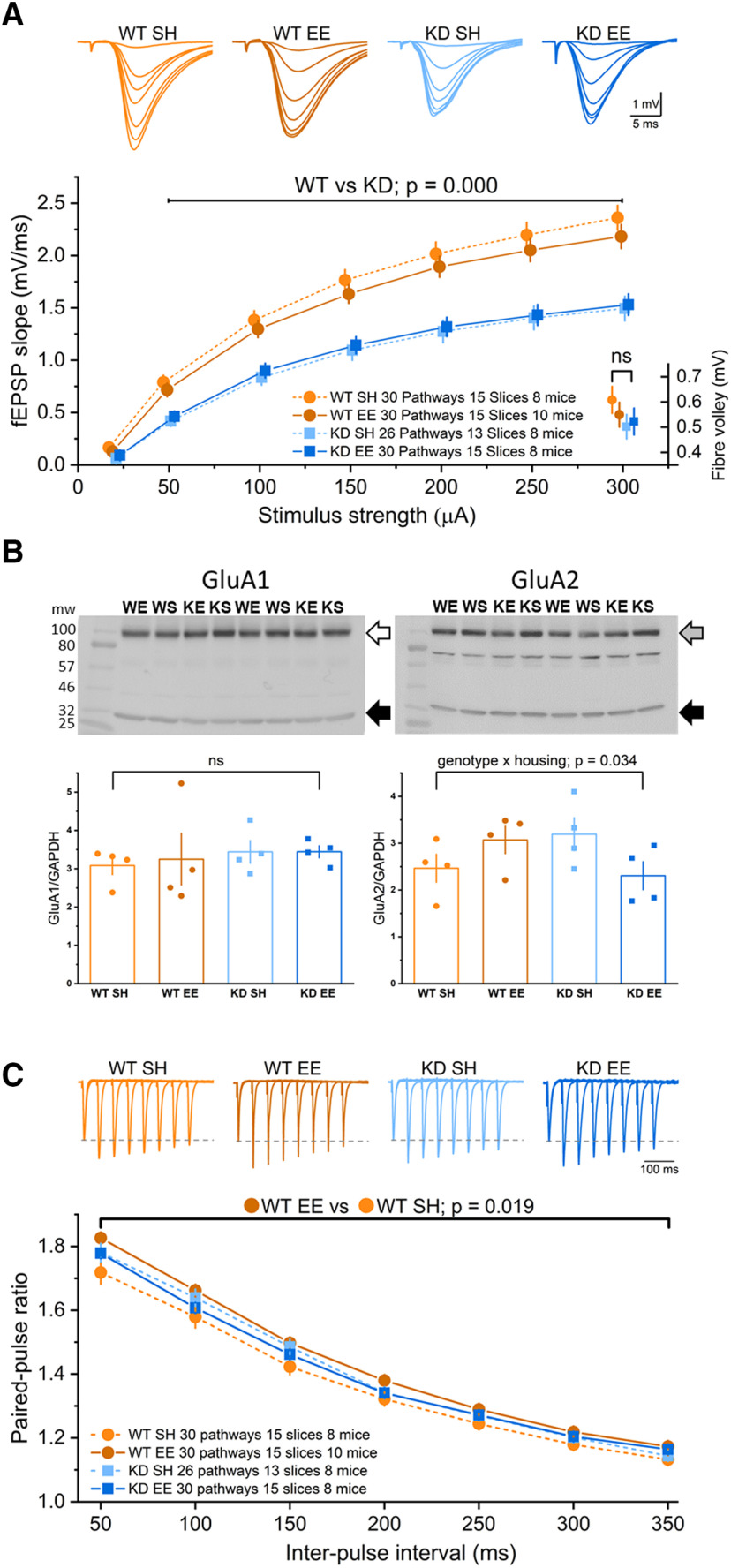
Enrichment has minimal effects on basal synaptic transmission, but differentially affects GluA2 expression and reduces the probability of neurotransmitter release in an MSK1-dependent manner. ***A***, *MSK1 KD* mice raised in standard (KD SH) and enriched (KD EE) housing displayed reduced synaptic transmission compared with WT mice (WT SH; WT EE), as previously described for standard housed WT and *MSK1 KD* mice (Daumas et al., 2017). A RM-ANOVA showed a significant effect of stimulus strength (*F*_(5,560)_ = 632.11, *p* = 0.000) and a significant effect of genotype (*F*_(1,112)_ = 43.99, *p* = 0.000), where the fEPSPs were larger in the WT mice regardless of their housing condition. Insets show representative fEPSPs at increasing stimulus strengths for each genotype and housing condition. Data are presented as mean ± SEM. Data points have been offset for clarity. ***B***, Western blots of GluA1 (left; open arrow) and GluA2 (right; gray arrow) expression showing two unique samples per blot from each of the four experimental groups. Quantification relative to GAPDH expression (filled arrow on blots) showed a significant genotype × housing interaction for GluA2 (*F*_(1,12)_ = 5.71, *p* = 0.034) but no significant results for GluA1 (ns: not statistically significant). One of two blots for each is shown. WT enriched, WE; WT standard housed, WS; *MSK1 KD* enriched, KE; *MSK1 KD* standard housed, KS. The ladder blot was digitally superimposed on the blots for GluA1, GluA2, and GAPDH. ***C***, There was no difference in PPF between standard housed WT and *MSK1 KD* mice. Equally, there were no differences between standard housed and enriched *MSK1 KD* mice. An RM-ANOVA showed a significant difference between standard housed and enriched WT mice, with enriched WT mice showing consistently greater PPF (*F*_(6,112)_ = 5.71, *p* = 0.019), consistent with a reduced initial probability of neurotransmitter release. Insets show representative PPF profiles for each genotype and housing condition, normalized to the amplitude of the first of each pair of fEPSPs (broken gray line). Data are presented as mean ± SEM.

To establish the extent to which these cognitive impairments were reflected at the cellular level, we performed electrophysiological recordings of basal excitatory synaptic transmission from area CA1 (Fig. 4). We have previously reported an enhancement in mEPSC amplitude after enrichment in WT, but not *MSK1 KD*, mice (Corrêa et al., 2012; Lalo et al., 2018). We also reported that mEPSCs in *MSK1 KD* mice were ∼10% smaller than those recorded from WT mice (Corrêa et al., 2012; Lalo et al., 2018) and more recently showed that this translated into smaller evoked field excitatory potentials (fEPSP) in area CA1 of *MSK1 KD* hippocampal slices (Daumas et al., 2017). Thus, to establish whether (1) environmental enrichment also enhanced synaptic transmission at the population fEPSP level in WT animals and (2) whether the basal fEPSP deficit in synaptic transmission in *MSK1 KD* mice persisted or was ameliorated after enrichment, we constructed input/output curves of stimulation strength versus the slope of the fEPSP in slices taken from animals raised under standard housing or environmentally-enriched housing (Fig. 4*A*).

Consistent with previous observations under standard housing conditions (Daumas et al., 2017), basal synaptic transmission in *MSK1 KD* slices was substantially reduced compared with that observed in WT slices (Fig. 4*A*). This was not due to an impairment in the recruitment of presynaptic axons as the fiber volley amplitudes (Fig. 4*A*) did not differ between these two groups, nor indeed between the groups having underwent enrichment. To establish whether the reduced synaptic transmission reflected differences in the expression of glutamate AMPA receptors, which are responsible for the majority of excitatory synaptic transmission at CA1 synapses, we conducted Western blotting for GluA1 and GluA2, the two primary AMPAR subunits contributing to synaptic transmission in area CA1 (Tsuzuki et al., 2001; Lu et al., 2009; Renner et al., 2017; Terashima et al., 2017; Diering and Huganir, 2018). No differences in GluA1 or GluA2 expression were observed under standard housing conditions that could explain the deficit in synaptic transmission observed in *MSK1 KD* mice (Fig. 4*B*).

The deficit in basal synaptic transmission in *MSK1 KD* mutant mice compared with WT mice persisted in animals raised in enriched conditions (Fig. 4*A*). Moreover, enrichment had no discernible effect on basal synaptic transmission in the *MSK1 KD* mutants; the input-output curves of standard housed and enriched *MSK1 KD* mice essentially overlapped. In contrast, synaptic transmission in slices from enriched WT mice was weaker over the entire range of stimulus strengths but did not reach statistical significance. While enrichment had no effect on GluA1 expression (Fig. 4*B*), there was an enrichment × genotype interaction for GluA2 levels such that there was an apparent increase and decrease in WT and *MSK1 KD* mice, respectively. These changes in GluA2 expression do not directly translate to the observed effects of enrichment on synaptic transmission, which saw decreases and no change in WT and *MSK1 KD* mice, respectively, but suggest that both experience and MSK1 can influence the expression of glutamate AMPA receptor subunits.

One potential explanation for the observation of a tendency to reduced synaptic transmission in WT mice is that there has been a reduction in the probability of evoked glutamate release, such that on average there are fewer synapses and postsynaptic AMPA receptors activated per stimulus. To test this, we constructed PPF profiles over the interpulse interval of 50–350 ms (Fig. 4*C*) as an index of the initial probability of glutamate release (Jackman and Regehr, 2017). Under standard housing conditions, and as reported previously (Daumas et al., 2017), the PPF profile of *MSK1 KD* mutant and WT slices were not different from one another. This suggests that MSK1 does not play a role in regulating transmitter release under standard housing conditions.

Similar to the lack of effect on basal synaptic transmission (Fig. 4*A*), raising *MSK1 KD* mice in enriched conditions had no effect on the PPF profile compared with their standard housed counterparts. However, WT mice raised in enriched housing showed a clear, consistent, and significant enhancement of PPF across the entire PPF range (Fig. 4*C*). Since PPF is inversely proportional to the initial probability of neurotransmitter release, this enhancement of PPF in WT slices likely reflects a reduction in the probability of glutamate release and may explain the consistent decrease in fEPSP strength observed in enriched WT mice (Fig. 4*A*). As such, these observations of changes in the probability of release in WT, but not MSK1 mutant hippocampal slices may be an adaptive, MSK1-dependent homeostatic response to the increase in mEPSCs we previously reported in enriched WT mice (Corrêa et al., 2012), potentially to limit network excitability.

**Figure 5.**
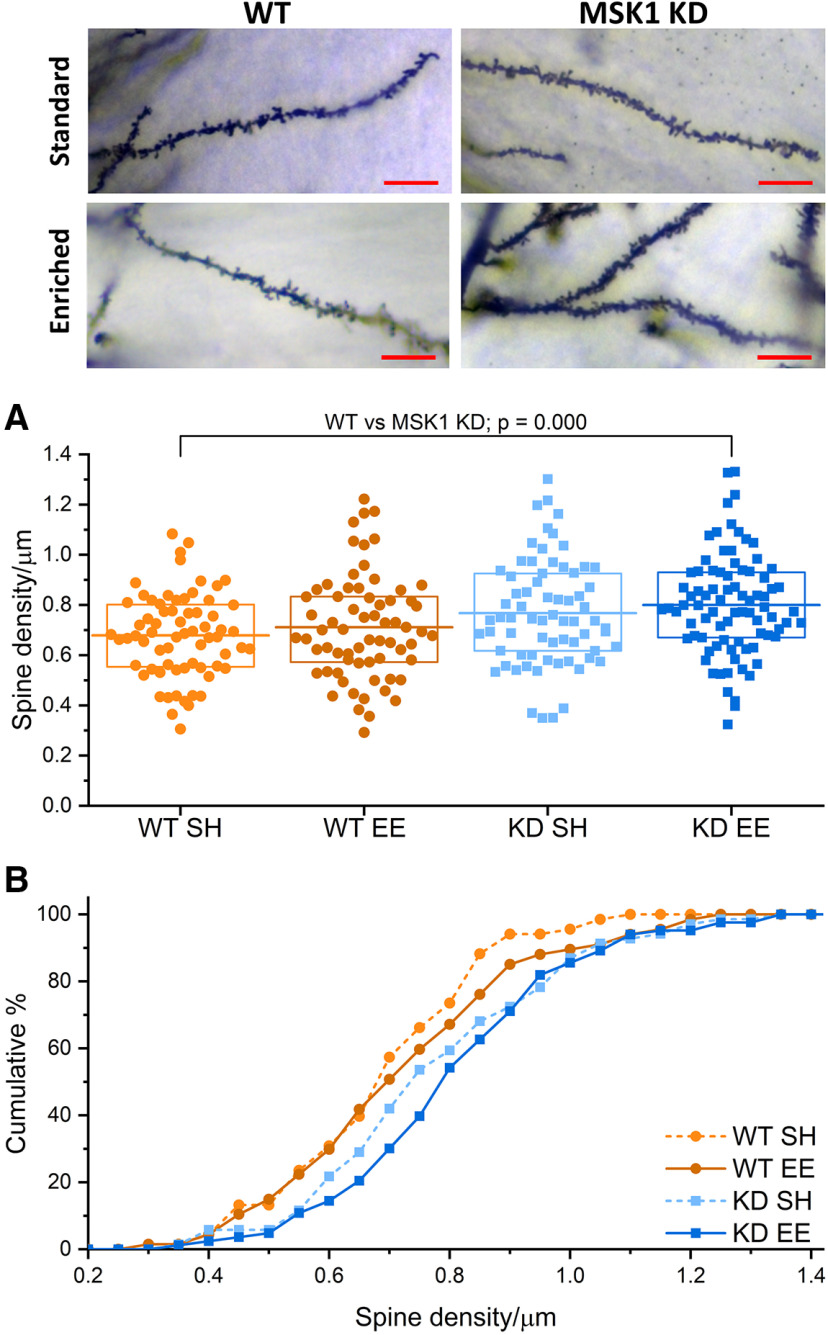
*MSK1 KD* mice have greater spine density than WT mice. ***A***, *MSK1 KD* mice had higher spine density than WT mice, consistent with observations made previously (Corrêa et al., 2012); genotype effect, *F*_(1,283)_ = 14.55, *p* = 0.000. Graph shows box plot of spine density distribution from 67–83 dendrites on CA1 neurons from four mice per condition. Box: 25–75% range; mean: horizontal line. Images show representative Golgi-stained CA1 secondary apical dendrites from across housing and genotype. Scale bars: 10 µm. ***B***, Graph shows cumulative distribution of spine density across housing and genotype. Note rightward shift in spine density distribution of *MSK1 KD* mutants (blue colors) reflecting the greater spine density shown in ***A***.

An alternative explanation, that the differences in synaptic transmission between genotypes and after enrichment in WT mice reflect corresponding decreases in the number of dendritic spines, is not supported by spine density measurements made from apical dendrites in Golgi-impregnated CA1 neurons (Fig. 5). As reported previously (Corrêa et al., 2012), CA1 apical dendrite spine density was significantly higher in *MSK1 KD* mice than in WT mice (Fig. 5*A*,*B*), with a trend toward greater spine density after enrichment in both genotypes.

### The experience-dependent enhancement of the dynamic range of synapses requires MSK1

These measures of basal synaptic transmission, the probability of glutamate release, GluA subunit expression, and spine density point to subtle cellular effects of enrichment that would not be expected to contribute appreciably to either the observed enhancement of cognition in WT mice, or the inability of *MSK1 KD* mice to display the full cognitive benefits of enrichment. Accordingly, to probe the potential cellular basis of the enrichment-dependent and MSK1-dependent enhancement of spatial learning and memory, we performed electrophysiological recordings of synaptic plasticity in area CA1 of hippocampal slices prepared from standard housed and enriched WT and *MSK1 KD* mutant mice.

**Figure 6.**
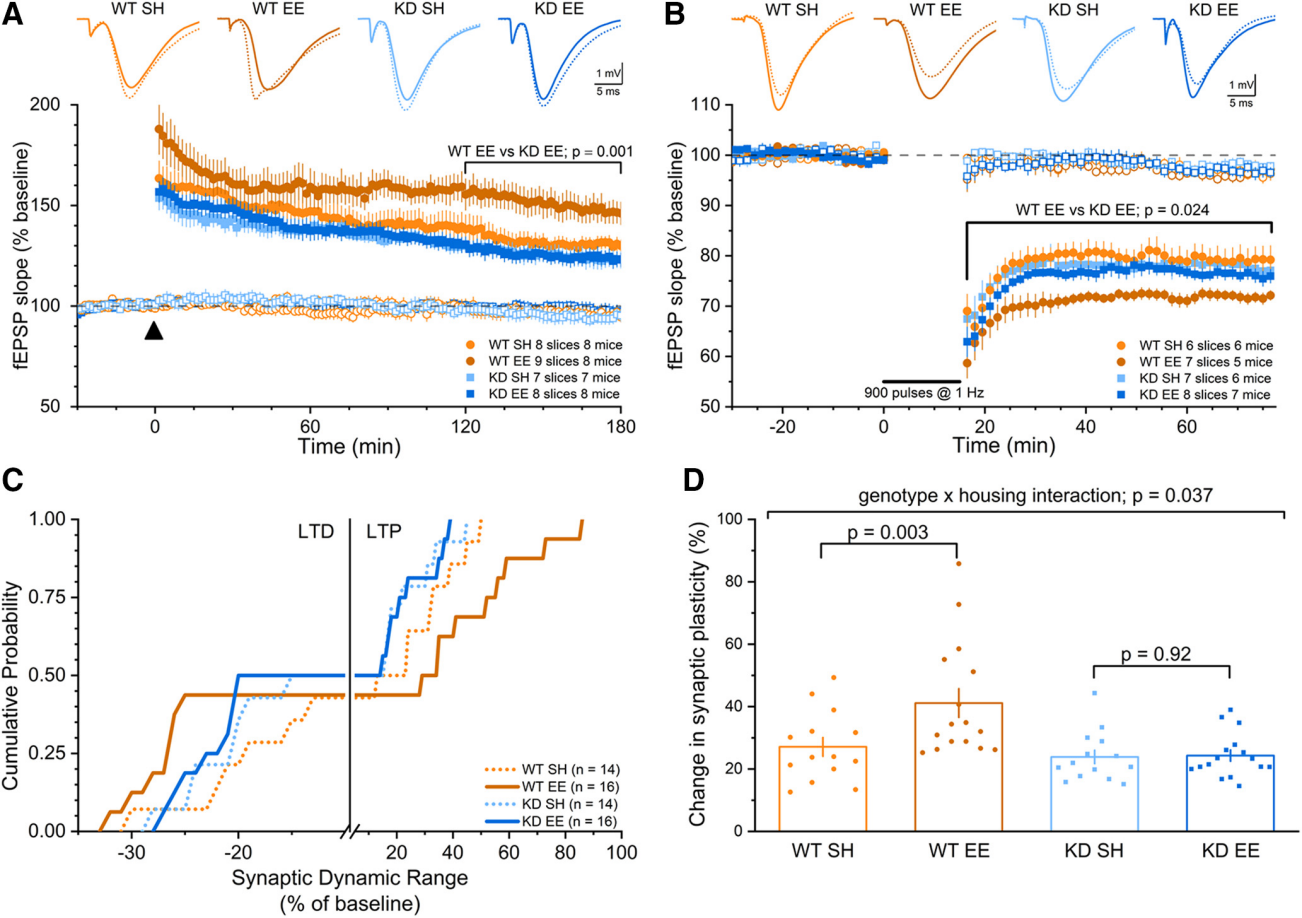
Experience enhances the dynamic range of synapses in an MSK1-dependent manner. ***A***, Robust LTP was induced by theta-burst stimulation in one pathway (at time 0, arrowhead; filled symbols), while the second pathway in each experiment (open symbols) remained unstimulated. LTP was enhanced exclusively in enriched WT slices. In the first 10 min after LTP induction an ANOVA revealed no appreciable genotype × housing interaction (*F*_(1,28)_ = 0.93, *p* = 0.343), but did show a significant effect of genotype (*F*_(1,28)_ = 4.78, *p* = 0.038). A *post hoc* analysis revealed LTP in the enriched WT group was significantly enhanced relative to enriched *MSK1 KD* mice (*F*_(1,28)_ = 5.29, *p* = 0.029). In the last hour of LTP (120–180 min), an RM-ANOVA revealed a strong trend for a genotype × housing interaction (*F*_(1,28)_ = 4.02, *p* = 0.055), in which LTP in the enriched WT group was significantly enhanced relative to enriched *MSK1 KD* mice (*F*_(1,28)_ = 11.16, *p* = 0.001) and standard housed WT mice (*F*_(1,28)_ = 8.37, *p* = 0.007). Insets are representative fEPSPs taken before (solid lines) and after (broken lines) LTP induction. Data shown as mean ± SEM. ***B***, At time 0, LTD was induced by delivering 900 pulses at 1 Hz to one pathway (filled symbols), while the second pathway in each experiment (open symbols) remained unstimulated. A significant genotype × housing interaction was observed (*F*_(1,24)_ = 4.47, *p* = 0.045), where LTD was enhanced in enriched WT mice relative to standard housed WT (*F*_(1,24)_ = 13.52, *p* = 0.001) and enriched *MSK1 KD* mice (*F*_(1,24)_ = 5.80, *p* = 0.024). Insets are representative fEPSPs taken before (solid lines) and after (broken lines) LTD induction. Data shown as mean ± SEM. ***C***, Cumulative distribution of changes of synaptic strength for each LTD and LTP experiment across housing and genotype. WT enriched mice show both leftward (LTD) and rightward (LTP) shifts in the range of synaptic depression and potentiation, respectively, compared with standard housed WT mice. The distributions for standard house and enriched *MSK1 KD* mice overlap indicating their insensitivity to enrichment. ***D***, The net extent of the change in synaptic strength from baseline values (100%) in each of the LTP (LTP% − 100%) and LTD(100% − LTD%) experiments shows a significant interaction between housing and genotype (*F*_(1,56)_ = 4.58, *p* = 0.037) and a significant effect of enrichment only in WT mice (*F*_(1,56)_ = 9.79, *p* = 0.003), and no effect in the *MSK1 KD* mutants (*F*_(1,56)_ = 0.10, *p* = 0.92), where the dynamic range of synaptic plasticity remains unchanged after enrichment. Bar graph depicts mean ± SEM and individual data points.

Since there is widespread agreement that activity-dependent changes in the efficacy of synaptic transmission, which have been observed after enrichment (Ohline and Abraham, 2019), underlie the ability of animals to learn and remember (Takeuchi et al., 2014), we predicted that: (1) enrichment would enhance the ability of synapses to display activity-dependent modifications of synaptic strength; and (2), given the impairment of cognition observed in MSK1 mutant mice, any enrichment-induced synaptic enhancement would require MSK1, and hence be absent in the MSK1 mutants. We thus performed dual-pathway LTP and LTD experiments from area CA1 in hippocampal slices from standard housed and enriched WT and *MSK1 KD* mice, where one pathway served as a time control, and the other pathway was subjected to plasticity-inducing stimulation. Consistent with our hypotheses, both LTP (Fig. 6*A*) and LTD (Fig. 6*B*) were significantly enhanced in the CA1 region of hippocampal slices prepared from WT mice that had received enrichment. In stark contrast, neither LTP nor LTD was affected by enrichment in the MSK1 mutant mice, where the extent of synaptic plasticity was comparable to that obtained from standard housed mice of both genotypes (Fig. 6*A*,*B*).

These observations indicate that MSK1 is required for the bidirectional expression of the enhanced plasticity associated with environmental enrichment, i.e., an expansion of the dynamic range of synapses, which occurred in the absence of appreciable changes in basal synaptic transmission or dendritic spine density (Figs. 4, 5), and in experiments where baseline fEPSPs were carefully stimulus matched for amplitude to take into account differences in basal synaptic transmission between *MSK1 KD* mutants and WT mice (see Materials and Methods: Hippocampal slice preparation and extracellular recordings).

To quantify the extent of the enrichment-dependent and MSK1-dependent expansion of the synaptic dynamic range, we compared, from the weakest to the strongest, the range of synaptic strengths recorded in the LTD and LTP experiments. On this basis, we calculated a ∼28% increase in the synaptic dynamic range in WT animals, but essentially no change in synaptic strength in response to enrichment in *MSK1 KD* mice (∼−5%). The bidirectional enhancement of synaptic strength in enriched WT animals can be appreciated in a plot of the cumulative distribution of individual LTD and LTP values for each of the experiments in each of the four groups of animals (Fig. 6*C*). A complementary comparison examined the net change from baseline (100%) in each of the LTP and LTD experiments (Fig. 6*D*) across both genotype and housing conditions. This analysis revealed that enrichment selectively enhanced the dynamic range of synapses in WT mice and, thus, demonstrates that the kinase activity of MSK1 is necessary for the experience-dependent bidirectional expansion of synaptic strength.

### Experience influences gene expression in anMSK1-dependent manner

**Figure 7.**
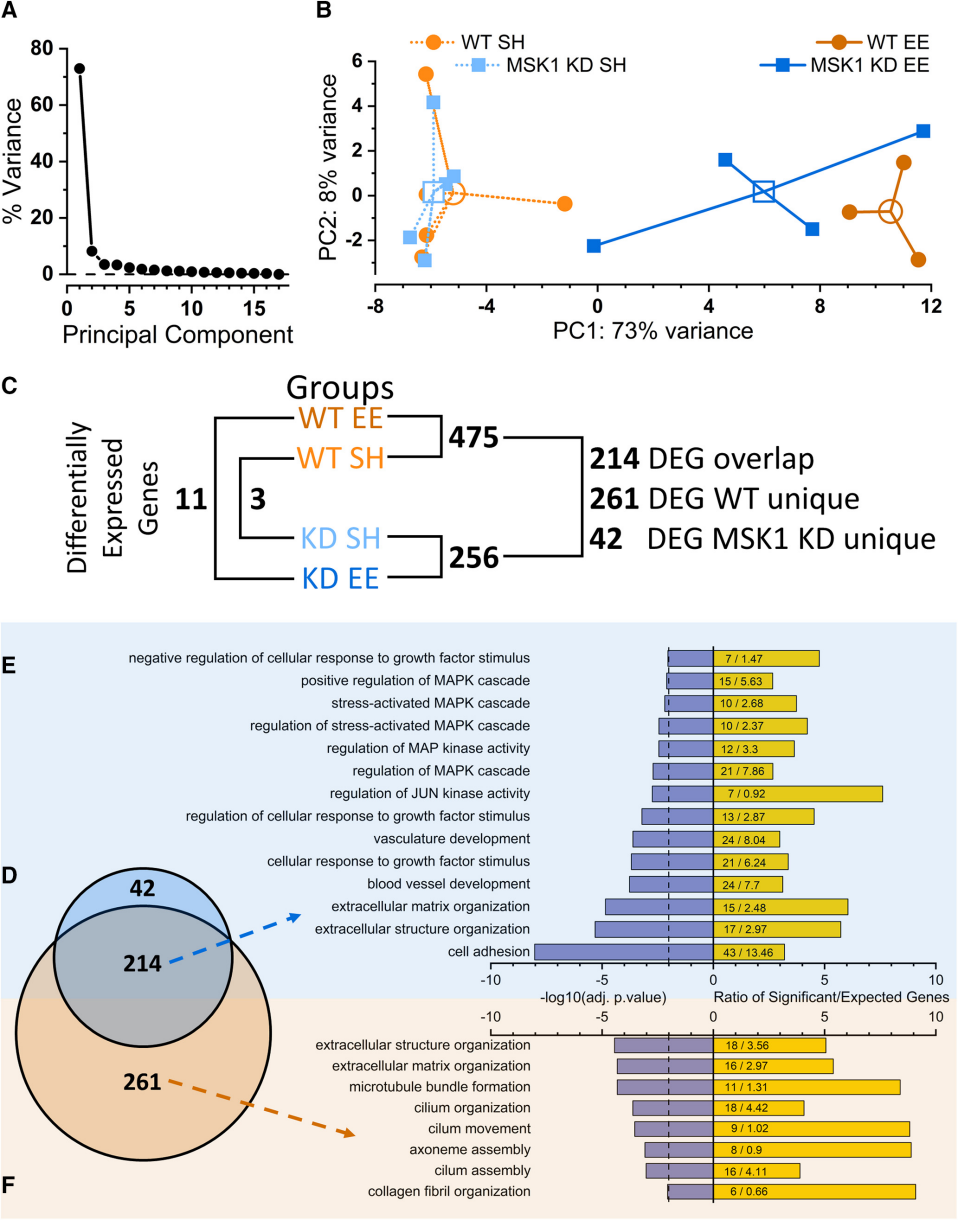
Environmental enrichment influences gene expression in an MSK1-dependent manner. ***A***, Scree plot of principal component (PC) percentage variation for top 500 variance transcripts. The majority of the variance is explained by one PC (73%), with the second PC contributing 8% of the variance. ***B***, PC analysis of top 500 transcripts by variation across samples (solid symbols). Experimental groups are distinguished by color. Each group (*n* = 3–5) is clustered around the arithmetic mean centroid (open symbol). The greatest variance (PC1; 73%) is explained by housing condition. ***C***, Schematic of differentially expressed gene (DEG) comparisons and number of DEGs between each genotype/housing condition combination (Extended Data Tables 7-3, 7-4, 7-5, 7-6, 7-7). ***D***, Venn diagram comparing DEGs in response to enriched environment versus standard housed controls. Orange circle: DEGs in WT mice exposed to EE (475 genes; Extended Data Table 7-11); cyan circle: DEGs in *MSK1 KD* mice exposed to EE (256 genes; Extended Data Table 7-12). ***E***, ***F***, Back-to-back bar plots of significantly enriched GO Terms against Benjamini-adjusted *p* values (lilac bars), and the ratio of significant genes contributing to each term to the number expected in each category (yellow bars; the actual values for the number of significant and expected genes for each category are given). Broken vertical line at −2 indicates *p* = 0.01. ***E***, Significantly enriched GO terms common to both WT and *MSK1 KD* mice exposed to enrichment (214 genes); 14 of 152 significant categories are shown (Extended Data Table 7-8). ***F***, Significantly enriched GO terms unique to WT mice exposed to enrichment (261 genes). Six of seven unique categories out of 10 significant categories are shown (Extended Data Table 7-9).

Given the dependence of persistent changes in synaptic function and cognition on gene expression (Alberini and Kandel, 2014), and the importance of MSK1 in regulating transcription (Reyskens and Arthur, 2016), including for key plasticity-related proteins such as Arc/Arg3.1 (Hunter et al., 2017), we addressed the molecular mechanisms downstream of MSK1 by examining patterns of gene expression in WT mice in which MSK1 was active and in kinase-dead animals in which it was not. RNA-Seq was performed on hippocampal tissue obtained from the four groups of mice. A principal component analysis (Fig. 7*A*,*B*) revealed that a striking 73% of the variance in gene expression was captured by the first principal component, corresponding to housing type, with both WT and *MSK1 KD* standard housed mice clustering together (Fig. 7*B*). The enriched WT group was readily distinguishable from these two groups, with members clustered tightly together. In contrast, the enriched *MSK1 KD* mice were distinct from both standard housed mice of both genotypes and enriched WT mice (Fig. 7*B*). Thus, enrichment had an effect on gene expression in *MSK1 KD* mice, but it appeared to be an uncoordinated or random response to enrichment, in stark contrast to the tightly regulated transcriptomic response in WT mice.

10.1523/JNEUROSCI.2765-19.2020.t7-4 Table 7-4. Supplementary Multimedia/Extended Data. Download Table 7-4, XLSX file

10.1523/JNEUROSCI.2765-19.2020.t7-5 Table 7-5. Supplementary Multimedia/Extended Data. Download Table 7-5, XLSX file

10.1523/JNEUROSCI.2765-19.2020.t7-6 Table 7-6. Supplementary Multimedia/Extended Data. Download Table 7-6, XLSX file

10.1523/JNEUROSCI.2765-19.2020.t7-7 Table 7-7. Supplementary Multimedia/Extended Data. Download Table 7-7, XLSX file

To identify the function of the genes regulated by enrichment, we performed GO analysis on genes differentially expressed in response to enrichment (Fig. 7*D–F*). The 214 MSK1-independent genes were distributed among 153 GO categories (Fig. 7*E*; Extended Data Table 7-8), which included cell adhesion, extracellular matrix, and structure organization and, notably, the regulation of mitogen-activated protein kinase (MAPK) signaling, of which MSK1 is an integral part. A similar GO analysis of the 261 unique MSK1-dependent genes revealed a restricted distribution among only 10 GO categories (Fig. 7*F*; Extended Data Table 7-9), with seven of these being unique to the MSK1-dependent genes. These unique categories encompassed microtubule bundle formation, cilium organization, assembly and movement, as well as ciliary axoneme assembly and collagen fibril organization.

10.1523/JNEUROSCI.2765-19.2020.t7-8 Table 7-8. Supplementary Multimedia/Extended Data. Download Table 7-8, XLSX file

10.1523/JNEUROSCI.2765-19.2020.t7-9 Table 7-9. Supplementary Multimedia/Extended Data. Download Table 7-9, XLSX file

10.1523/JNEUROSCI.2765-19.2020.t7-10 Table 7-10. Supplementary Multimedia/Extended Data. Download Table 7-10, XLSX file

10.1523/JNEUROSCI.2765-19.2020.t7-11 Table 7-11. Supplementary Multimedia/Extended Data. Download Table 7-11, XLSX file

10.1523/JNEUROSCI.2765-19.2020.t7-12 Table 7-12. Supplementary Multimedia/Extended Data. Download Table 7-12, XLSX file

Differential gene expression analysis revealed that, while there were only three differentially-expressed genes between standard housed WT and *MSK1 KD* mice (Fig. 7*C*; Extended Data Tables 7-3, 7-4), enrichment affected the regulation of 261 unique genes in WT mice (Fig. 7*C*,*D*; Extended Data Table 7-5). In contrast, only 42 genes showed selective regulation by enrichment in the *MSK1 KD* mice (Fig. 7*C*,*D*; Extended Data Table 7-6), with an additional 214 genes regulated by enrichment in both WT and *MSK1 KD* mice (Fig. 7*C*,*D*; Extended Data Table 7-7). These data indicate that the majority of genes affected by enrichment are regulated by MSK1.

### MSK1 is necessary for an experience-dependent homeostatic downregulation of plasticity gene expression

**Figure 8.**
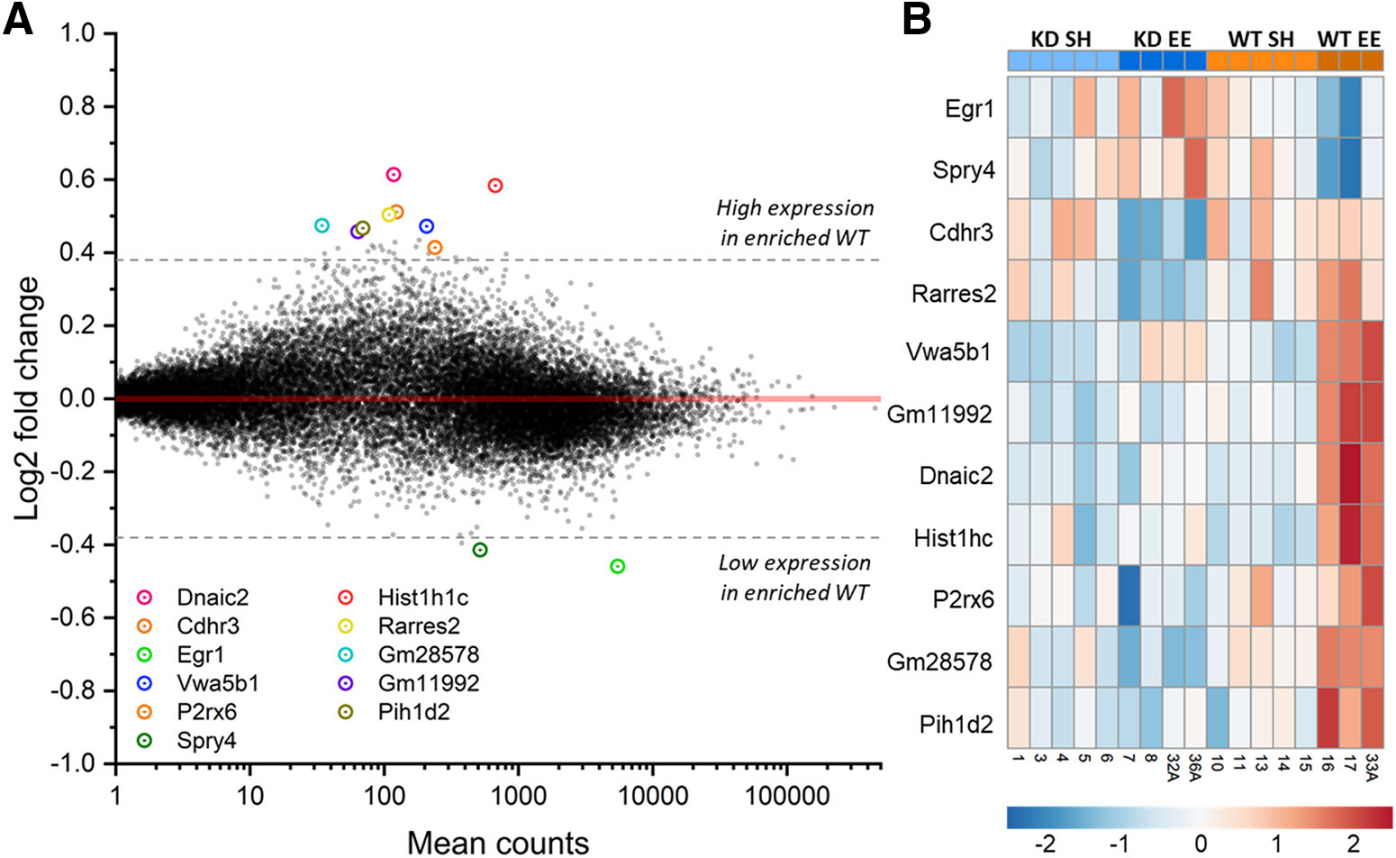
Enrichment downregulates neurotrophin signaling-related genes in a MSK1-dependent manner. ***A***, MA plot of environmentally-enriched WT versus environmentally-enriched *MSK1 KD* groups. DESeq2 β-prior transformed log2 fold-changes are plotted on the *y*-axis. Broken lines at ±0.38 equate to a ±1.3-fold change. Significantly differentially expressed genes are circled and labeled. ***B***, Heatmap of Z-score normalized rlog expression for genes identified as significantly different between *MSK1 KD* enriched (KD EE) and WT enriched (WT EE) groups (Extended Data Table 8-10). Red cells indicate overexpression, blue cells indicate underexpression of a gene in a sample. Colors and labels at the top and bottom of the heatmap correspond to the sample's housing and genotype identity.

**Figure 9.**
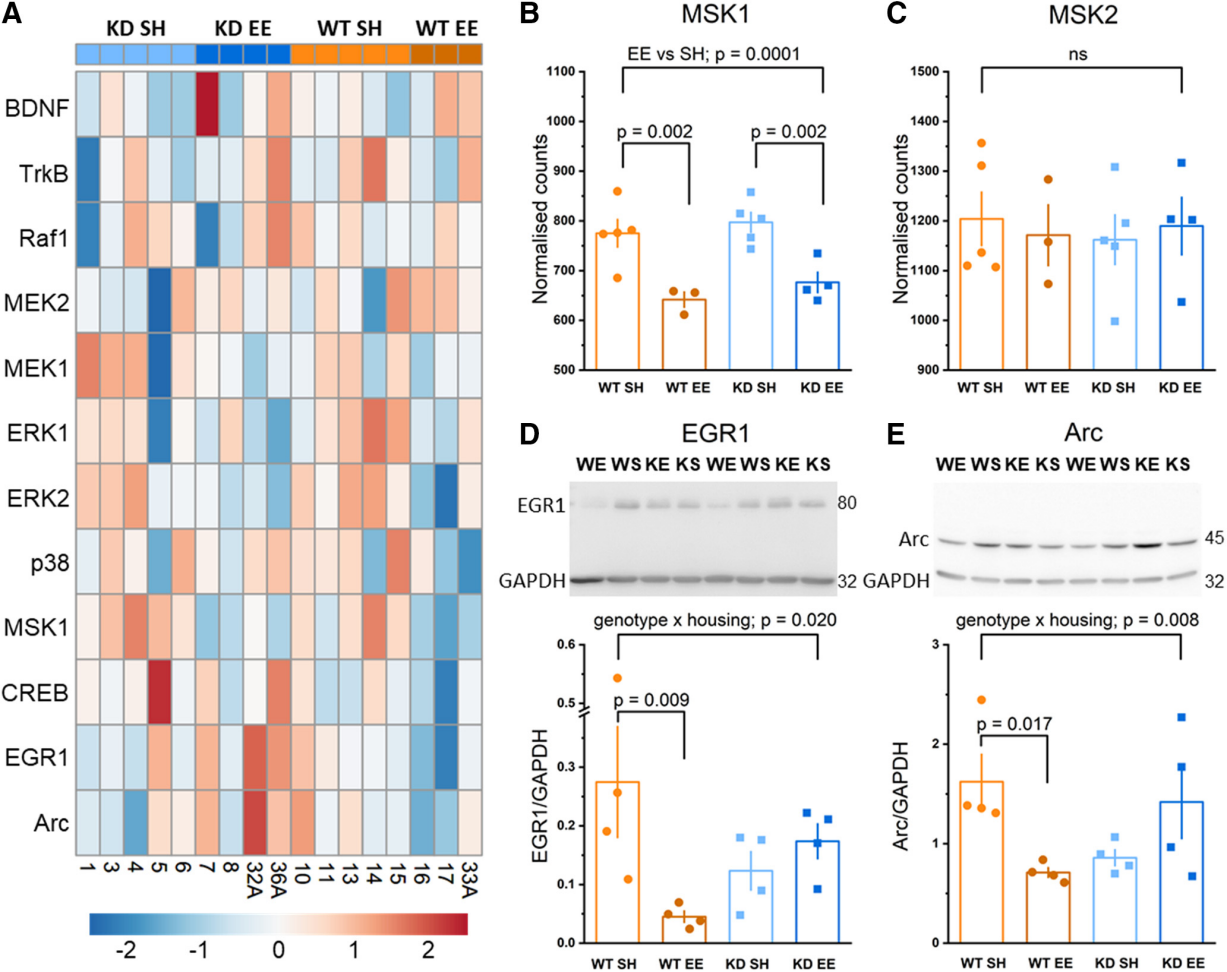
MSK1 orchestrates homeostatic genomic downscaling of plasticity-related pathways and proteins in response to enrichment. ***A***, Heatmap of genes relevant to the BDNF/MAPK/MSK1 signaling cascade showing selective downregulation in samples from enriched WT mice (WT EE). For clarity, the protein name is provided. For where the gene and protein name are not the same: TrkB2, Ntrk2; MEK2, Map2k2; MEK1, Map2k1; ERK1, Mapk3; ERK2, Mapk1; p38, Mapk14; MSK1, Rps6ka5; CREB, Creb1. ***B***, ***C***, The expression of MSK1, but not MSK2, is selectively downregulated in response to enrichment (standard vs enriched housing; two-way ANOVA; *F*_(3,13)_ = 29.01, *p* = 0.0001) in both WT (*F*_(1,13)_ = 14.74, *p* = 0.002) and *MSK1 KD* mice (*F*_(1,13)_ = 14.31, *p* = 0.002). Bar graph depicts mean ± SEM and individual data points; ns: not statistically significant. ***D***, Western blot analysis of EGR1 protein in hippocampal tissue from separate samples contemporaneous with those used for RNA-Seq. There was a significant interaction genotype × housing; *F*_(3,12)_ = 7.17, *p* = 0.020 for EGR1 protein expression, which was selectively strongly downregulated in WT enriched mice (*F*_(1,12)_ = 9.63, *p* = 0.009), in keeping with RNA-Seq DEG analysis (Fig. 8*A*,*B*). Inset shows one of the two blots used in the analysis of EGR1 expression. WE, WT enriched (*n* = 4); WS, WT standard housed (*n* = 4); KE, *MSK1 KD* enriched (*n* = 4); KS, *MSK1 KD* standard housed (*n* = 4). Bar graph depicts mean ± SEM and individual data points. ***E***, Western blot analysis of Arc/Arg3.1 protein in hippocampal tissue from separate samples contemporaneous with those used for RNA-Seq. There was a significant interaction genotype × housing; *F*_(3,12)_ = 9.95, *p* = 0.008 for Arc/Arg3.1 protein expression, which was selectively strongly downregulated in WT enriched mice (*F*_(1,12)_ = 7.64, *p* = 0.017). Inset shows one of the two blots used in the analysis of Arc/Arg3.1 expression. WE, WT enriched (*n* = 4); WS, WT standard housed (*n* = 4); KE, *MSK1 KD* enriched (*n* = 4); KS, *MSK1 KD* standard housed (*n* = 4). Bar graph depicts mean ± SEM and individual data points.

An examination of specific genes regulated by both MSK1 and enrichment revealed 11 genes (Fig. 8*A*,*B*; Extended Data Table 8-10) that were significantly differently expressed between enriched WT and *MSK1 KD* mutant mice. In particular, two genes regulated by neurotrophins and the MAPK cascade were strongly downregulated in enriched WT mice: Sprouty4 (Spry4; Cabrita and Christofori, 2008) and early growth response protein 1 (Egr1/Zif268/NGFI-A; Veyrac et al., 2014; Duclot and Kabbaj, 2017). This unexpected downregulation of Egr1, which has repeatedly been shown to be elevated acutely in response to enrichment, plasticity-inducing, and learning and memory-inducing stimuli (Pinaud, 2004; Veyrac et al., 2014; Duclot and Kabbaj, 2017), prompted a curated investigation of genes relevant to the activation of MSK1. This analysis (Fig. 9*A*) revealed a striking and unexpected enrichment-induced downregulation of the MSK1 signaling cascade, including of MSK1 itself (Fig. 9*B*), but not of the related MSK2 isoform (Fig. 9*C*). In addition to downregulation of Egr1, downregulation was also observed of the MSK1 substrate CREB, and of the key plasticity-related protein Arc/Arg3.1 (Epstein and Finkbeiner, 2018), which we have previously shown was regulated in an MSK1-dependent manner during both homeostatic synaptic plasticity (Corrêa et al., 2012) and in response to BDNF (Hunter et al., 2017). That this enrichment-induced reduction of MSK1 gene expression had tangible effects on Egr1 and Arc/Arg3.1 protein expression was confirmed by Western blots from contemporaneous hippocampal tissue, which showed strong enrichment-induced downregulation of Egr1 and Arc/Arg3.1 protein levels exclusively in WT mice (Fig. 9*D*,*E*). These data indicate that MSK1 orchestrates an experience-dependent homeostatic downregulation of key plasticity-related proteins.

10.1523/JNEUROSCI.2765-19.2020.t8-10 Table 8-10. Supplementary Multimedia/Extended Data. Download Table 8-10, XLSX file

## Discussion

The molecular pathway that links the external environment to the genomic response that underpins experience-dependent neuronal and cognitive adaptations has remained elusive, but is important to identify given the potential for pharmacological manipulation to remediate the cognitive deficits associated with congenital, acquired and age-related cognitive impairment (Guerrieri et al., 2017; Consorti et al., 2019). While both BDNF and a range of plasticity-related proteins have been implicated in the neuronal response to enrichment (Cowansage et al., 2010; Sale et al., 2014; Rogers et al., 2019), the mechanism that allows experience to couple BDNF to plasticity-related proteins has yet to be described.

### MSK1 orchestrates an experience-dependent genomic homeostasis

We have shown previously that mice lacking the kinase activity of MSK1, an enzyme regulated by BDNF (Arthur et al., 2004; Daumas et al., 2017; Hunter et al., 2017), fail to upregulate miniature excitatory synaptic currents in response to either enrichment *in vivo*, or activity deprivation *in vitro* (Corrêa et al., 2012; Lalo et al., 2018). This selective mutation of the MSK1 gene obviates concerns regarding a structural role for MSK1 (Gutièrrez-Mecinas et al., 2011) that may contribute to the basal (Chwang et al., 2007; Choi et al., 2012; Karelina et al., 2012) and enrichment-induced (Karelina et al., 2012) deficits in learning and memory and neurogenesis observed in mice in which MSK1 has been constitutively deleted. Instead, the *MSK1 KD* mutation allows the influence of experience to be examined against an intact basal synaptic plasticity and cognitive repertoire (Daumas et al., 2017).

Using mice lacking the kinase activity of MSK1, we have presented evidence from several independent, but convergent lines of investigation, from genes to behavior, that MSK1 is a strong candidate for acting as an important link between the environment and the genome. While we have used male mice, which predominate in environmental enrichment research (Simpson and Kelly, 2011; Girbovan and Plamondon, 2013), it is unlikely that our observations would not generalize to females given the reported increases in BDNF production and MAPK activity in female rodents after enrichment (Bakos et al., 2009; Ramírez-Rodríguez et al., 2014). However, this should be empirically tested in subsequent studies.

Through comparing WT and *MSK1 KD* mice, we have shown that the kinase activity of MSK1 has an obligatory role in the regulation of the transcriptional response to experience, the modulation of the dynamic range of synapses, the persistence of memory, and cognitive flexibility. We propose that these effects are causally linked. A coordinated and MSK1-dependent pattern of gene expression likely facilitates the growth and development of the enriched brain, with Spry4 and genes regulating the primary cilium being especially targeted. Spry4 exerts an inhibitory influence on the actions of neurotrophins (Cabrita and Christofori, 2008; Alsina et al., 2012) and on axonal outgrowth (Hausott et al., 2012). The experience-dependent downregulation of Spry4 would thus be expected to remove this inhibition allowing greater influence of neurotrophins on neuronal structure and function. The primary cilium is a cellular organelle that protrudes from the surface of virtually all mammalian cells, including neurons, and has important signaling properties (Nachury and Mick, 2019). The cilium plays an important role in dendritic arborization (Guadiana et al., 2013), neuronal development, and neurogenesis (Guemez-Gamboa et al., 2014; Valente et al., 2014; Lepanto et al., 2016), and has been implicated in the maturation of neuronal circuits, synaptic plasticity, and learning and memory (Kumamoto et al., 2012; Rhee et al., 2016). These, and other, MSK1-dependent genes are therefore likely to contribute to the frequently observed changes in brain structure and function that support experience-dependent enhanced cognition (Rosenzweig and Leiman, 1968; Rosenzweig and Bennett, 1996; Kolb and Whishaw, 1998). Moreover, once having facilitated the functional and structural response to enrichment, MSK1 orchestrates a genomic homeostatic scaling characterized by the downregulation of the transcription factor EGR1 and the plasticity-related protein Arc/Arg3.1. The function of this unexpected downregulation, which is also observed in upstream MSK1-activating kinases such as ERK2 and P38, and indeed of MSK1 itself, may be to stabilize and preserve the neuronal networks, synaptic plasticity, and cognitive enhancement arising from experience. Thus, the normal pattern of MSK1-dependent gene expression can be homeostatically down-tuned in response to enrichment in WT mice, but this adaptive ability is lost in the *MSK1 KD* mice, likely resulting in the genomic, synaptic, and cognitive impairments seen in the mutants.

### MSK1 regulates the dynamic range of synapses and is required for the full expression of experience-dependent enhancement of cognition

In parallel, experience augments the dynamic range of hippocampal synapses in an MSK1-dependent manner through the enhancement of both LTP and LTD. MSK1 thus enables synapses to both store more information and potentially be more responsive to prevailing synaptic and neuronal activity. This may manifest as the ability to rapidly switch from one learned behavior, potentially by both weakening established neuronal circuits in an LTD-like manner, and mastering another, and for longer, through LTP-like strengthening of new networks. Hippocampus-dependent behavioral correlates of these forms of synaptic plasticity, reversal learning, and the persistence of spatial memory were both impaired in mice lacking the kinase activity of MSK1. This suggests that the MSK1-dependent expansion of the dynamic range of synapses increases the information capacity of synapses, underpins the experience-dependent enhancement of cognition, and thus provides a plausible mechanism for the consistent improvements in cognition repeatedly observed in response to enrichment since their first description in the 1940s by Donald Hebb (Hebb, 1947, 1949). Equally, the increased number of dendritic spines in *MSK1 KD* mice observed here and in a previous study (Corrêa et al., 2012) suggest that MSK1 influences spine density, either constitutively as a regulator of gene expression or in an activity-dependent manner in response to synaptic activity. This dysregulation of spine number in the *MSK1 KD* mutant may contribute in particular to the cognitive impairments seen in *MSK1 KD* mice after enrichment and has parallels with the greater spine density and impaired cognition observed in both human autism spectrum disorder and animal models of autism (Coley and Gao, 2018; Nakai et al., 2018). MSK1 may thus coordinate the neuronal mechanisms and networks supporting synaptic structure, function, plasticity, and cognition through the regulation of gene expression. After enrichment, the MSK1-driven genomic downregulation of plasticity-related proteins leads to a reduced baseline against which activity-dependent elevations in their transcription may proportionally have greater synaptic and cognitive impact.

### MSK1 is a key regulator of experience-dependent and activity-dependent genomic, synaptic and cognitive plasticity

The recruitment of MSK1 during exposure to a complex environment underpins a range of adaptive genomic, molecular, and synaptic responses that contribute appreciably to the experience-dependent enhancement of cognition. Moreover, by initiating the downregulation of key plasticity-related genes, MSK1 plays a pivotal role in ensuring the stability of the new and improved experience-dependent genomic, neuronal, and cognitive landscape, and one that is potentially primed to respond more effectively to new challenges. MSK1, from regulating homeostatic synaptic scaling *in vitro* (Corrêa et al., 2012) to initiating genomic homeostasis in response to experience *in vivo*, thus represents a key molecular sensor linking the environment and prevailing synaptic activity to the genome and the ensuing adaptive neuronal and cognitive response.

## References

[B1] Alberini CM, Kandel ER (2014) The regulation of transcription in memory consolidation. Cold Spring Harb Perspect Biol 7:a021741. 10.1101/cshperspect.a021741 25475090 PMC4292167

[B2] Alexa A, Rahnenfuhrer J (2006) topGO: enrichment analysis for gene ontology. R package version 2.32.0 Available at https://bioconductor.org/packages/release/bioc/html/topGO.html.

[B3] Alsina FC, Irala D, Fontanet PA, Hita FJ, Ledda F, Paratcha G (2012) Sprouty4 is an endogenous negative modulator of TrkA signaling and neuronal differentiation induced by NGF. PLoS One 7:e32087. 10.1371/journal.pone.0032087 22384148 PMC3285629

[B4] Anders S, Pyl PT, Huber W (2015) HTSeq–a Python framework to work with high-throughput sequencing data. Bioinformatics 31:166–169. 10.1093/bioinformatics/btu638 25260700 PMC4287950

[B5] Anderson WW, Collingridge GL (2007) Capabilities of the WinLTP data acquisition program extending beyond basic LTP experimental functions. J Neurosci Methods 162:346–356. 10.1016/j.jneumeth.2006.12.018 17306885

[B6] Andrews S (2010) FastQC: a quality control tool for high throughput sequence data. Available at http://www.bioinformatics.babraham.ac.uk/projects/fastqc/.

[B7] Andrews S (2018) SeqMonk: a tool to visualise and analyse high throughput mapped sequence data. Available at https://www.bioinformatics.babraham.ac.uk/projects/seqmonk/.

[B8] Aronoff E, Hillyer R, Leon M (2016) Environmental enrichment therapy for autism: outcomes with increased access. Neural Plast 2016:2734915. 10.1155/2016/2734915 27721995 PMC5046013

[B9] Arthur JS, Fong AL, Dwyer JM, Davare M, Reese E, Obrietan K, Impey S (2004) Mitogen- and stress-activated protein kinase 1 mediates cAMP response element-binding protein phosphorylation and activation by neurotrophins. J Neurosci 24:4324–4332. 15128846 10.1523/JNEUROSCI.5227-03.2004 PMC6729446

[B10] Bakos J, Hlavacova N, Rajman M, Ondicova K, Koros C, Kitraki E, Steinbusch HW, Jezova D (2009) Enriched environment influences hormonal status and hippocampal brain derived neurotrophic factor in a sex dependent manner. Neuroscience 164:788–797. 10.1016/j.neuroscience.2009.08.054 19723563

[B11] Cabrita MA, Christofori G (2008) Sprouty proteins, masterminds of receptor tyrosine kinase signaling. Angiogenesis 11:53–62. 10.1007/s10456-008-9089-1 18219583

[B12] Carlson M (2018) org.Mm.eg.db: genome wide annotation for mouse. R package version 3.6.0. Available at https://bioconductor.org/packages/release/data/annotation/html/org.Mm.eg.db.html.

[B13] Choi YS, Karelina K, zate-Correa D, Hoyt KR, Impey S, Simon AJ, Obrietan K (2012) Mitogen- and stress-activated kinases regulate progenitor cell proliferation and neuron development in the adult dentate gyrus. J Neurochem 123:676–688. 23020821 10.1111/jnc.12035 PMC3575744

[B14] Chwang WB, Arthur JS, Schumacher A, Sweatt JD (2007) The nuclear kinase mitogen- and stress-activated protein kinase 1 regulates hippocampal chromatin remodeling in memory formation. J Neurosci 27:12732–12742. 18003853 10.1523/JNEUROSCI.2522-07.2007 PMC5724774

[B15] Coley AA, Gao WJ (2018) PSD95: a synaptic protein implicated in schizophrenia or autism? Prog Neuropsychopharmacol Biol Psychiatry 82:187–194. 10.1016/j.pnpbp.2017.11.016 29169997 PMC5801047

[B16] Consorti A, Sansevero G, Torelli C, Berardi N, Sale A (2019) From basic visual science to neurodevelopmental disorders: the voyage of environmental enrichment-like stimulation. Neural Plast 2019:5653180. 10.1155/2019/5653180 31198418 PMC6526521

[B17] Corrêa SA, Hunter CJ, Palygin O, Wauters SC, Martin KJ, McKenzie C, McKelvey K, Morris RG, Pankratov Y, Arthur JS, Frenguelli BG (2012) MSK1 regulates homeostatic and experience-dependent synaptic plasticity. J Neurosci 32:13039–13051. 10.1523/JNEUROSCI.0930-12.2012 22993422 PMC6621478

[B18] Cowansage KK, Ledoux JE, Monfils MH (2010) Brain-derived neurotrophic factor: a dynamic gatekeeper of neural plasticity. Curr Mol Pharmacol 3:12–29. 10.2174/1874467211003010012 20030625

[B19] D'Amato FR, Zanettini C, Sgobio C, Sarli C, Carone V, Moles A, Ammassari-Teule M (2011) Intensification of maternal care by double-mothering boosts cognitive function and hippocampal morphology in the adult offspring. Hippocampus 21:298–308. 10.1002/hipo.20750 20087885

[B20] Daumas S, Hunter CJ, Mistry RB, Morè L, Privitera L, Cooper DD, Reyskens KM, Flynn HT, Morris RG, Arthur JS, Frenguelli BG (2017) The kinase function of MSK1 regulates BDNF signaling to CREB and basal synaptic transmission, but is not required for hippocampal long-term potentiation or spatial memory. eNeuro 4:ENEURO.0212-16.2017 10.1523/ENEURO.0212-16.2017 PMC5318545 28275711

[B21] de la Tremblaye PB, Cheng JP, Bondi CO, Kline AE (2019) Environmental enrichment, alone or in combination with various pharmacotherapies, confers marked benefits after traumatic brain injury. Neuropharmacology 145:13–24. 10.1016/j.neuropharm.2018.02.032 29499273

[B22] Diering GH, Huganir RL (2018) The AMPA receptor code of synaptic plasticity. Neuron 100:314–329. 10.1016/j.neuron.2018.10.018 30359599 PMC6214363

[B23] Dobin A, Davis CA, Schlesinger F, Drenkow J, Zaleski C, Jha S, Batut P, Chaisson M, Gingeras TR (2013) STAR: ultrafast universal RNA-seq aligner. Bioinformatics 29:15–21. 10.1093/bioinformatics/bts635 23104886 PMC3530905

[B24] Duclot F, Kabbaj M (2017) The role of early growth response 1 (EGR1) in brain plasticity and neuropsychiatric disorders. Front Behav Neurosci 11:35. 10.3389/fnbeh.2017.00035 28321184 PMC5337695

[B25] Epstein I, Finkbeiner S (2018) The arc of cognition: signaling cascades regulating Arc and implications for cognitive function and disease. Semin Cell Dev Biol 77:63–72. 10.1016/j.semcdb.2017.09.023 29559111 PMC5865643

[B26] Farah MJ (2018) Socioeconomic status and the brain: prospects for neuroscience-informed policy. Nat Rev Neurosci 19:428–438. 10.1038/s41583-018-0023-2 29867123

[B27] Faraway JJ (2005) Linear models with R. Boca Raton: Chapman and Hall/CRC.

[B28] Girbovan C, Plamondon H (2013) Environmental enrichment in female rodents: considerations in the effects on behavior and biochemical markers. Behav Brain Res 253:178–190. 10.1016/j.bbr.2013.07.018 23860119

[B29] Guadiana SM, Semple-Rowland S, Daroszewski D, Madorsky I, Breunig JJ, Mykytyn K, Sarkisian MR (2013) Arborization of dendrites by developing neocortical neurons is dependent on primary cilia and type 3 adenylyl cyclase. J Neurosci 33:2626–2638. 10.1523/JNEUROSCI.2906-12.2013 23392690 PMC6619186

[B30] Gubert C, Hannan AJ (2019) Environmental enrichment as an experience-dependent modulator of social plasticity and cognition. Brain Res 1717:1–14. 10.1016/j.brainres.2019.03.033 30930150

[B31] Guemez-Gamboa A, Coufal NG, Gleeson JG (2014) Primary cilia in the developing and mature brain. Neuron 82:511–521. 10.1016/j.neuron.2014.04.024 24811376 PMC4104280

[B32] Guerrieri D, Moon HY, van Praag H (2017) Exercise in a pill: the latest on exercise-mimetics. Brain Plast 2:153–169. 10.3233/BPL-160043 29765854 PMC5928571

[B33] Gutièrrez-Mecinas M, Trollope AF, Collins A, Morfett H, Hesketh SA, Kersanté F, Reul JMHM (2011) Long-lasting behavioral responses to stress involve a direct interaction of glucocorticoid receptors with ERK1/2-MSK1-Elk-1 signaling. Proc Natl Acad Sci USA 108:13806–13811. 10.1073/pnas.1104383108 21808001 PMC3158237

[B34] Hausott B, Vallant N, Schlick B, Auer M, Nimmervoll B, Obermair GJ, Schwarzer C, Dai F, Brand-Saberi B, Klimaschewski L (2012) Sprouty2 and -4 regulate axon outgrowth by hippocampal neurons. Hippocampus 22:434–441. 10.1002/hipo.20910 21240919

[B35] Hebb DO (1947) The effects of early experience on problem-solving at maturity. Am Psychol 2:306–307.

[B36] Hebb DO (1949) The organization of behaviour. New York: Wiley.

[B37] Heffron D, Mandell JW (2005) Differential localization of MAPK-activated protein kinases RSK1 and MSK1 in mouse brain. Brain Res Mol Brain Res 136:134–141. 10.1016/j.molbrainres.2005.01.014 15893597

[B38] Hirase H, Shinohara Y (2014) Transformation of cortical and hippocampal neural circuit by environmental enrichment. Neuroscience 280:282–298. 10.1016/j.neuroscience.2014.09.031 25242640

[B39] Howell DC (2010) Statistical methods for psychology, Ed 7 Belmont: Thomson Wadsworth.

[B40] Hunter CJ, Remenyi J, Correa SA, Privitera L, Reyskens KM, Martin KJ, Toth R, Frenguelli BG, Arthur JS (2017) MSK1 regulates transcriptional induction of Arc/Arg3.1 in response to neurotrophins. FEBS Open Bio 7:821–834. 10.1002/2211-5463.12232 PMC5458472 28593137

[B41] Jackman SL, Regehr WG (2017) The mechanisms and functions of synaptic facilitation. Neuron 94:447–464. 10.1016/j.neuron.2017.02.047 28472650 PMC5865607

[B42] Jax (2009) A Jackson Laboratory resource manual: breeding strategies for maintaining colonies of laboratory mice. Available at https://www.research.uci.edu/forms/docs/iacuc/JAX-breeding-strategies.pdf.

[B43] Jiang H, Lei R, Ding SW, Zhu S (2014) Skewer: a fast and accurate adapter trimmer for next-generation sequencing paired-end reads. BMC Bioinformatics 15:182. 10.1186/1471-2105-15-182 24925680 PMC4074385

[B44] Karelina K, Hansen KF, Choi YS, DeVries AC, Arthur JS, Obrietan K (2012) MSK1 regulates environmental enrichment-induced hippocampal plasticity and cognitive enhancement. LearnMem 19:550–560. 10.1101/lm.025775.112 PMC3475155 23077336

[B45] Kolb B, Whishaw IQ (1998) Brain plasticity and behavior. Annu Rev Psychol 49:43–64. 10.1146/annurev.psych.49.1.43 9496621

[B46] Kumamoto N, Gu Y, Wang J, Janoschka S, Takemaru K, Levine J, Ge S (2012) A role for primary cilia in glutamatergic synaptic integration of adult-born neurons. Nat Neurosci 15:399–405, S1. 10.1038/nn.3042 22306608 PMC3288565

[B47] Kutner MH, Nachtsheim CJ, Neter J, Li W (2005) Applied linear statistical models, Ed 5 Boston: McGraw-Hill Irwin.

[B48] Laerd Statistics (2017) Two-way ANOVA using SPSS statistics. Statistical tutorials and software guides. Available at https://statistics.laerd.com/.

[B49] Lalo U, Bogdanov A, Moss GW, Frenguelli BG, Pankratov Y (2018) Role for astroglia-derived BDNF and MSK1 in homeostatic synaptic plasticity. Neuroglia 1:381–394. 10.3390/neuroglia1020026

[B50] Lepanto P, Badano JL, Zolessi FR (2016) Neuron's little helper: the role of primary cilia in neurogenesis. Neurogenesis (Austin) 3:e1253363. 10.1080/23262133.2016.1253363 28090545 PMC5129898

[B51] Lipp HP, Wolfer DP (1998) Genetically modified mice and cognition. Curr Opin Neurobiol 8:272–280. 10.1016/s0959-4388(98)80151-7 9635213

[B52] Love MI, Huber W, Anders S (2014) Moderated estimation of fold change and dispersion for RNA-seq data with DESeq2. Genome Biol 15:550. 10.1186/s13059-014-0550-8 25516281 PMC4302049

[B53] Lu W, Shi Y, Jackson AC, Bjorgan K, During MJ, Sprengel R, Seeburg PH, Nicoll RA (2009) Subunit composition of synaptic AMPA receptors revealed by a single-cell genetic approach. Neuron 62:254–268. 10.1016/j.neuron.2009.02.027 19409270 PMC3632349

[B54] Miguel PM, Pereira LO, Silveira PP, Meaney MJ (2019) Early environmental influences on the development of children's brain structure and function. Dev Med Child Neurol 61:1127–1133. 10.1111/dmcn.14182 30740660

[B55] Mo C, Hannan AJ, Renoir T (2015) Environmental factors as modulators of neurodegeneration: insights from gene-environment interactions in Huntington's disease. Neurosci Biobehav Rev 52:178–192. 10.1016/j.neubiorev.2015.03.003 25770041

[B56] Mohler EG, Shacham S, Noiman S, Lezoualc'h F, Robert S, Gastineau M, Rutkowski J, Marantz Y, Dumuis A, Bockaert J, Gold PE, Ragozzino ME (2007) VRX-03011, a novel 5-HT4 agonist, enhances memory and hippocampal acetylcholine efflux. Neuropharmacology 53:563–573. 10.1016/j.neuropharm.2007.06.016 17692343

[B57] Nachury MV, Mick DU (2019) Establishing and regulating the composition of cilia for signal transduction. Nat Rev Mol Cell Biol 20:389–405. 10.1038/s41580-019-0116-4 30948801 PMC6738346

[B58] Nakai N, Takumi T, Nakai J, Sato M (2018) Common defects of spine dynamics and circuit function in neurodevelopmental disorders: a systematic review of findings from in vivo optical imaging of mouse models. Front Neurosci 12:412. 10.3389/fnins.2018.00412 29970983 PMC6018076

[B59] Ohline SM, Abraham WC (2019) Environmental enrichment effects onsynaptic and cellular physiology of hippocampal neurons. Neuropharmacology 145:3–12. 10.1016/j.neuropharm.2018.04.007 29634984

[B60] Pinaud R (2004) Experience-dependent immediate early gene expression in the adult central nervous system: evidence from enriched-environment studies. Int J Neurosci 114:321–333. 10.1080/00207450490264142 14754658

[B61] Ramírez-Rodríguez G, Ocaña-Fernández MA, Vega-Rivera NM, Torres-Pérez OM, Gómez-Sánchez A, Estrada-Camarena E, Ortiz-López L (2014) Environmental enrichment induces neuroplastic changes in middle age female Balb/c mice and increases the hippocampal levels of BDNF, p-Akt and p-MAPK1/2. Neuroscience 260:158–170. 10.1016/j.neuroscience.2013.12.026 24361917

[B62] Renner MC, Albers EH, Gutierrez-Castellanos N, Reinders NR, van Huijstee AN, Xiong H, Lodder TR, Kessels HW (2017) Synaptic plasticity through activation of GluA3-containing AMPA-receptors. Elife 6:e25462 10.7554/eLife.25462 28762944 PMC5578739

[B63] Reyskens KM, Arthur JS (2016) Emerging roles of the mitogen and stress activated kinases MSK1 and MSK2. Front Cell Dev Biol 4:56. 10.3389/fcell.2016.00056 27376065 PMC4901046

[B64] Rhee S, Kirschen GW, Gu Y, Ge S (2016) Depletion of primary cilia from mature dentate granule cells impairs hippocampus-dependent contextual memory. Sci Rep 6:34370. 10.1038/srep34370 27678193 PMC5039642

[B65] Robinson JT, Thorvaldsdóttir H, Winckler W, Guttman M, Lander ES, Getz G, Mesirov JP (2011) Integrative genomics viewer. Nat Biotechnol 29:24–26. 10.1038/nbt.1754 21221095 PMC3346182

[B66] Rogers J, Renoir T, Hannan AJ (2019) Gene-environment interactions informing therapeutic approaches to cognitive and affective disorders. Neuropharmacology 145:37–48. 10.1016/j.neuropharm.2017.12.038 29277490

[B67] Rosenzweig MR, Leiman AL (1968) Brain functions. Annu Rev Psychol 19:55–98. 10.1146/annurev.ps.19.020168.000415 4866842

[B68] Rosenzweig MR, Bennett EL (1996) Psychobiology of plasticity: effects of training and experience on brain and behavior. Behav Brain Res 78:57–65. 10.1016/0166-4328(95)00216-2 8793038

[B69] RTeam (2018) R: a language and environment for statistical computing. Available at www.R-project.org.

[B70] Sale A, Berardi N, Maffei L (2014) Environment and brain plasticity: towards an endogenous pharmacotherapy. Physiol Rev 94:189–234. 10.1152/physrev.00036.2012 24382886

[B71] Simpson J, Kelly JP (2011) The impact of environmental enrichment in laboratory rats–behavioural and neurochemical aspects. Behav Brain Res 222:246–264. 10.1016/j.bbr.2011.04.002 21504762

[B72] Sindreu CB, Scheiner ZS, Storm DR (2007) Ca2+ -stimulated adenylyl cyclases regulate ERK-dependent activation of MSK1 during fear conditioning. Neuron 53:79–89. 10.1016/j.neuron.2006.11.024 17196532 PMC1858648

[B73] Stairs DJ, Bardo MT (2009) Neurobehavioral effects of environmental enrichment and drug abuse vulnerability. Pharmacol Biochem Behav 92:377–382. 10.1016/j.pbb.2009.01.016 19463254 PMC2687322

[B74] Takeuchi T, Duszkiewicz AJ, Morris RG (2014) The synaptic plasticity and memory hypothesis: encoding, storage and persistence. Philos Trans R Soc Lond B Biol Sci 369:20130288. 10.1098/rstb.2013.0288 24298167 PMC3843897

[B75] Terashima A, Suh YH, Isaac JTR (2017) The AMPA receptor subunit GluA1 is required for CA1 hippocampal long-term potentiation but is not essential for synaptic transmission. 44:549–561. 10.1007/s11064-017-2425-3 29098531

[B76] Tsuzuki K, Lambolez B, Rossier J, Ozawa S (2001) Absolute quantification of AMPA receptor subunit mRNAs in single hippocampal neurons. J Neurochem 77:1650–1659. 10.1046/j.1471-4159.2001.00388.x 11413248

[B77] Valente EM, Rosti RO, Gibbs E, Gleeson JG (2014) Primary cilia in neurodevelopmental disorders. Nat Rev Neurol 10:27–36. 10.1038/nrneurol.2013.247 24296655 PMC3989897

[B78] Veyrac A, Besnard A, Caboche J, Davis S, Laroche S (2014) The transcription factor Zif268/Egr1, brain plasticity, and memory. Prog Mol Biol Transl Sci 122:89–129. 10.1016/B978-0-12-420170-5.00004-0 24484699

[B79] von Bernhardi R, Eugenín-von-Bernhardi L, Eugenín J (2017) What is neural plasticity? Adv Exp Med Biol 1015:1–15. 10.1007/978-3-319-62817-2_1 29080018

[B80] Wassouf Z, Schulze-Hentrich JM (2019) Alpha-synuclein at the nexus of genes and environment: the impact of environmental enrichment and stress on brain health and disease. J Neurochem 150:591–604. 10.1111/jnc.14787 31165472 PMC6771760

[B81] Wei J, Carroll RJ, Harden KK, Wu G (2012) Comparisons of treatment means when factors do not interact in two-factorial studies. Amino Acids 42:2031–2035. 10.1007/s00726-011-0924-0 21547361 PMC3199378

[B82] Wolf LW, LaRegina MC, Tolbert DL (1996) A behavioral study of the development of hereditary cerebellar ataxia in the shaker rat mutant. Behav Brain Res 75:67–81. 10.1016/0166-4328(96)00159-3 8800661

[B83] Woo CC, Leon M (2013) Environmental enrichment as an effective treatment for autism: a randomized controlled trial. Behav Neurosci 127:487–497. 10.1037/a0033010 23688137

[B84] Woo CC, Donnelly JH, Steinberg-Epstein R, Leon M (2015) Environmental enrichment as a therapy for autism: a clinical trial replication and extension. Behav Neurosci 129:412–422. 10.1037/bne0000068 26052790 PMC4682896

